# Printing technologies for monitoring crop health

**DOI:** 10.1038/s41467-026-68778-6

**Published:** 2026-01-24

**Authors:** David Panáček, Vojtěch Kupka, Martin-Alex Nalepa, Ivan Dědek, Ruslan Álvarez-Diduk, Selin Olenik, Jose Flauzino, Jan Zdražil, Petr Jakubec, Lukáš Zdražil, Lukáš Spíchal, Keval K. Sonigara, Radek Zbořil, Martin Pumera, Arben Merkoçi, Joseph Wang, Nuria De Diego, Firat Güder, Michal Otyepka

**Affiliations:** 1 Department of Bioengineering, Royal School of Mines, Imperial College London, London, UK; 2 Czech Advanced Technology and Research Institute (CATRIN), Palacký University Olomouc, Olomouc, Czechia; 3 Department of Physical and Macromolecular Chemistry, Faculty of Science, Charles University, Prague, Czechia; 4 Catalan Institute of Nanoscience and Nanotechnology, ICN2, BIST, and CSIC, Campus UAB, Bellaterra, Barcelona, Spain; 5 Bezos Centre for Sustainable Protein, Imperial College London, London, UK; 6 Faculty of Electrical Engineering and Computer Science, VSB-Technical University of Ostrava, Ostrava, Czechia; 7 Center for Advanced Technologies and Engineering (CATEN), Ostrava-Pustkovec, Czechia; 8 Nanotechnology Centre, Centre of Energy and Environmental Technologies, VŠB–Technical University of Ostrava, Ostrava-Poruba, Czechia; 9 Future Energy and Innovation Laboratory, Central European Institute of Technology, Brno University of Technology, Brno, Czechia; 10 Catalan Institution for Research and Advanced Studies (ICREA), Barcelona, Spain; 11 Aiiso Yufeng Li Family Department of Chemical and Nano Engineering, University of California San Diego, La Jolla, CA USA; 12 Centre for Processable Electronics, Imperial College London, London, UK; 13 IT4Innovations, VŠB-Technical University of Ostrava, Ostrava-Poruba, Czechia

**Keywords:** Plant stress responses, Sensors, Sensors and probes

## Abstract

Agricultural production requires low-cost sensors capable of delivering reliable, high-resolution data across large areas. Rising food demand, limited arable land, and severe soil degradation have accelerated the adoption of precision agriculture, which relies on real-time monitoring of soil, plant, and environmental conditions. Central to this shift is the development of scalable sensor technologies enabled by advances in materials science. Printing techniques, including inkjet, screen, aerosol jet, 3D printing, and direct laser writing, offer versatile routes to fabricate flexible, large-area, and plant-integrated sensors. This Review surveys recent progress in printable low-dimensional materials for agricultural sensing, examines their physicochemical properties in relation to sensor performance, and discusses key challenges and future opportunities requiring interdisciplinary integration.

## Abstract (web summary)

Precision agriculture requires scalable, low-cost sensor technologies capable of delivering real-time, high-resolution insights into soil, plant, and environmental conditions. Here, the authors review printable materials and devices in enabling real-time and continuous monitoring for smart agriculture.

## Introduction

Global agriculture is under increasing pressure to meet the growing demand for food while dealing with climate variability, resource limitations, and stricter sustainability regulations^1^. As a pillar of modern society, agriculture has undergone extensive industrial and market-driven transformations, leading to higher productivity but also introducing pressing environmental challenges worldwide. Recent geopolitical shifts and socio-economic trends underscore the key role of agriculture in food systems, economic development, and global stability. Simultaneously, approximately 40% of the Earth’s land area is dedicated to agriculture, while only 11% is considered cultivable and suitable for crop production^2^. Productive land and fertile soil remain our most critical nonrenewable geo-resources. Erosion and pollution caused by humans, however, lead to the depletion of 24 billion tonnes of fertile soil each year, causing an annual economic loss of approximately USD 490 billion^3^. In this context, securing food supplies requires integrated strategies that enhance productivity while preserving environmental integrity^4–7^.

**Fig. 1:**
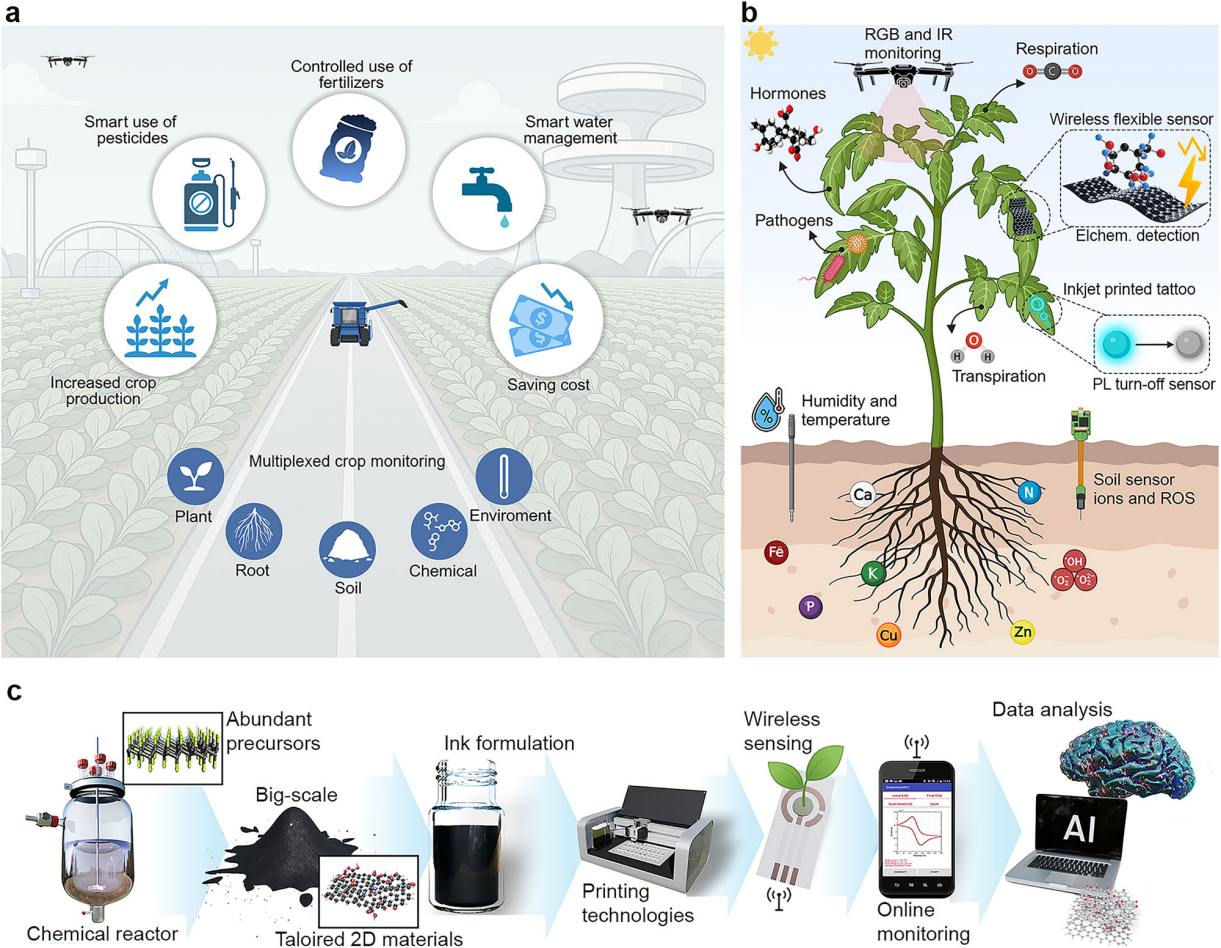
Illustration of the benefits and future trends in next-generation plant monitoring. **a** A schematic of precision agriculture showing the foundational benefits of modern farming systems. By integrating digital technologies, precision agriculture enables real-time monitoring, smart and controlled nutrition, pesticide and water management, thus contributing to increased crop productivity and reduced resource and material costs. **b** An image of an advanced smart plant monitoring illustrating the future of next-generation diagnostics through diverse sensing modalities, including multimodal and wearable sensor platforms. **c** Workflow toward manufacturing and utilization of printed sensor, including low-dimensional material synthesis, ink formulation, printing, deployment, and data processing. **a** was created in BioRender. Alharthi, A. (2026) https://BioRender.com/utumi3x. **b** was created in BioRender. Alharthi, A. (2026) https://BioRender.com/zebch33.

A promising approach to address these challenges is the integration of precision agriculture (Fig. 1a) with AI-driven data processing for more accurate decision support^8–10^. This enables precise control for targeted delivery of resources (fertilizers, pesticides, and irrigation water), thereby increasing crop yields, saving cost, and minimizing environmental impact^11–13^. The importance of real-time and on-site data on soil properties (moisture, pH, and nutrient composition)^14^, plant physiology (hormone levels, stress markers)^15^, and environmental conditions (temperature, light intensity, relative humidity (RH), and CO_2_ concentration)^16^ is essential for optimizing agricultural management^4,5,17,18^. Agricultural applications require sensors capable of monitoring key parameters, such as soil nutrients, moisture, salinity, pH, and leaf-level indicators, including humidity/VPD, metabolites, and stress biomarkers. These measurements are taken across diverse environments, including soil, leaves, stems, fruits, irrigation lines, and both open-field and greenhouse systems. Printed and biodegradable sensors are well-positioned to meet these needs by enabling continuous, distributed monitoring, which supports timely decisions in irrigation, fertilization, and plant stress management.

Printing technologies offer exactly what agriculture needs: low-cost, flexible, and biodegradable sensors that can be produced at scale and deployed anywhere on the plant or in the field. This makes printed devices strong candidates for overcoming the practical limitations of current sensing tools. Traditional approaches, including manual sampling for laboratory analysis and visual crop inspection, are slow, labor-intensive, and often inaccurate. While visual observation can be automated through plant imaging, it typically detects symptoms only after physiological stress has already progressed and the first stress signals have been activated, thereby hindering timely decision-making^19^. Plants rapidly respond to dynamic stressors (Fig. 1b) by the emission or accumulation of chemical signals, such as ion fluxes^20^, small radicals [e.g., reactive oxygen species (ROS)^21^], volatile organic compounds (VOCs) (e.g., methanol, acetic acid, methyl jasmonate)^22^ or phytohormones^23^, over timescales ranging from seconds to days^24^. Specific fast-occurring signals, such as superoxide radicals, occur at precise moments, serving as concrete signals that activate a plant response cascade and may be missed with low and insufficient sampling frequency^25^. Although conventional methods, such as leaf-disc punching^26^, freeze extraction^27^, or root exudate sampling^28^ are widely used in plant monitoring, they become unsuitable for repeated real-time measurements and may even alter the physiological processes studied^29^. These invasive assays are also incompatible with field deployment because they require controlled handling, cold-chain transport, and benchtop instrumentation, which are impractical across distributed plots and can miss the critical moment when the plant senses changing environmental conditions. Consequently, advancements in sensor technology that continuously monitor plant biochemical changes prioritize continuous, nondestructive measurement approaches^14,15,30^. Constant monitoring of these dynamic signals, rather than discrete interval sampling, can enable researchers and growers to gain deeper insight into plant defense mechanisms and identify emerging threats on time. In response, autonomous electrochemical and optical sensor systems are increasingly implemented, providing continuous monitoring and enabling rapid, data-driven interventions in real-time, thus minimizing substantial damage and reducing costs^31–35^.

Among these autonomous systems, electrochemical sensors represent a prominent class of devices that enable selective detection and quantification of analytes when integrated with conductive and electrochemically active advanced materials^36,37^. Sensors can be directly integrated into soil^38^ and plant tissues^39^ or attached to leaves^40,41^ for continuous monitoring of ions^14^, ROS^40^, and metabolites^39^. Optical sensors offer additional opportunities and, compared to electrochemical sensors, provide a complementary signal that can be effectively utilized for applications in real-time monitoring^42,43^. Optical sensors use a noninvasive detection mechanism (e.g., drone technology^44^, see Fig. 1b) and seamless integration with systems, such as infrared sensing^45^, fluorescence monitoring^46^, or RGB imaging^43,47^, make them an effective alternative to electrochemical detection. Optical sensors, however, face significant challenges, including delayed responses in capturing the plant’s current state, since physiological changes often appear in optical observations much later than in electrochemical readouts^48,49^. Although both electrochemical (fast response, high sensitivity) and optical (noninvasive detection, smooth integration into real-world applications) sensors exhibit significant potential for plant monitoring, many currently market-available systems fall short in practical agricultural applications.

Despite recent advancements, current commercial plant sensors are limited to basic environmental metrics, such as soil moisture, temperature, humidity, and light. A major limitation of current commercial plant-monitoring systems is their reliance on conventional electronic components (their architectures do not incorporate printed electronics) and simple resistive measurement principles, which are optimized for soil-level parameters rather than direct leaf or stem interface. They often lack precision and remain too costly (ranging from USD 100 to USD 2000 per device) for large-scale, multi-point agricultural monitoring deployment. Crucially, these devices lack functional materials. They are unable to detect key physiological markers, such as specific ions, phytohormones, or other signaling molecules, which are critical for precision agriculture and early-warning diagnostics. Examples of such commercially available systems include the Xiaomi Mi Flora Monitor, Willow Plant Sensor, FYTA Beam, EarthOne, and Sonoff for consumer applications, as well as agricultural-grade sensors, such as Sensoterra, Niubo, CropX, Arable, and METOS, which similarly focus on soil parameters rather than plant biochemical status. This gap underscores the urgent need for advances in materials development, signal transduction, data processing, and sensor durability to ensure reliable performance under variable and fluctuating environmental conditions. Addressing these limitations requires not only innovation in sensor hardware but also the integration of predictive modeling techniques, such as machine learning (ML), with real-time data acquisition. Such integration can transform raw sensor outputs into actionable insights, enabling early detection of stress signals and allowing farmers to implement timely, targeted interventions guided by data-driven decision-making^50–52^.

Among various platforms for agricultural monitoring, printed sensors stand out for their unique potential to meet the scalability, cost-efficiency, and deployment demands of precision agriculture^14,15,38–40^. Although many reported demonstrations rely on non-crop species, chosen as model plants because their large and mechanically robust leaves facilitate early prototyping of printed and inkjet-printed devices, the underlying sensing principles and fabrication strategies remain directly transferable to crop-relevant systems. By using additive manufacturing techniques, including screen printing (SP)^14,53^, inkjet printing (IJP)^54,55^, 3D printing (3DP)^40,56^, aerosol jet printing (AJP)^57,58^, or direct laser writing (DLW)^59,60^, printed sensors reduce overall costs and shorten production cycles compared to conventional methods, such as photolithography and vacuum deposition^61,62^. These devices can be easily integrated on flexible^63–66^ or biodegradable^67–69^ substrates, enabling environmentally friendly deployment and facilitating scalable production of advanced sensor arrays optimized for real-world agricultural applications (Fig. 1c). Moreover, advances in the development of functional electrochemically active inks containing metal nanoparticles^70–73^, polymers^74–76^, carbon^77–79^, or biomolecules^80–82^ are further expanding the portfolio of applications in sensor design. However, several challenges must be addressed, including excessive material consumption (SP, 3DP)^83,84^ and significant irreversible losses during electrode fabrication due to ink retention on the mesh, stencil, and squeegee, particularly in SP^85^. In addition, optimizing print compatibility with many substrates is still demanding, which is one of the main drawbacks of IJP^86,87^. Despite all the challenges, the dynamic and rapidly evolving field of printing technology has great potential to meet all the demands of smart agriculture by improving production processes, minimizing or eliminating redundant steps in conventional methods, and facilitating rapid prototyping to provide robust, sensitive, and adaptable sensors (Fig. 1c). For realistic agricultural use, sensors must be robust against environmental stressors, such as temperature variation, sunlight, moisture, and plant movement. Encapsulated ultrathin printed devices, as demonstrated in recent studies, already show stable multi-day operation on living leaves. Calibration and signal stability are essential due to fluctuating field conditions, while biodegradable substrates allow tunable device lifetimes with minimal environmental impact. Integration with wireless data systems and simple readout electronics enables real-time processing of plant information for automated management.

Furthermore, these methods have significant promise beyond agricultural monitoring, extending to wearable, biomedical, and environmental sensing systems. This review, however, concentrates specifically on their implementation and adaptation for crop and plant monitoring, where biocompatibility, conformability, and environmental resilience are critical. While several reviews have discussed printed electronics or biosensors in general, none have provided a unified comparison of all major printing technologies specifically in the context of plant and agricultural monitoring. This review bridges that gap by integrating screen, inkjet, 3D, aerosol jet, and laser-based printing within a single framework, highlighting their unique advantages, limitations, and complementarities for scalable, sustainable, and real-world sensing applications.

This review explores the emerging role of printing technologies in enabling real-time and continuous monitoring for smart agriculture. We begin by outlining printing methods specifically developed for plant science applications, emphasizing their potential for scalable, rapid, and customizable sensor fabrication. A comprehensive analysis of key additive manufacturing techniques follows, including their respective advantages, limitations, and future directions. We also examine the critical physicochemical properties of printable inks, such as viscosity, surface tension, particle morphology, and postprocessing requirements, which directly influence print quality and device performance. The review further investigates the potential of advanced low-dimensional materials, including MXenes, transition metal dichalcogenides (TMDs), and graphene derivatives, as promising ink platforms for plant monitoring. In conclusion, we highlight emerging trends and emphasize the importance of an integrated interdisciplinary approach that connects plant science, materials engineering, and data analytics to drive innovations and deliver impactful solutions on a global scale.

## Printing technologies

Printing technology, in the context of electronic and sensor development, refers to the additive patterning of functional materials onto a wide variety of substrates using digitally or physically guided deposition methods. Unlike traditional subtractive techniques, such as photolithography, which require multistep processes involving masking, etching, and cleanroom environments with UV or electron beam exposure^88^, printing offers a more cost-effective, scalable, and environmentally friendly alternative^61,89^. One of the defining advantages of printing technologies is their broad substrate compatibility. Devices can be printed on both conventional materials, such as plastics^90^, textiles^91^, and glass^92^, or biodegradable substrates^93–95^, including cellulose paper^96^, and even natural plant surfaces, such as leaves and stems^57,97,98^. This versatility supports the development of sustainable, field-deployable sensors that can naturally degrade after use, reducing agricultural waste and aligning with the principles of the circular economy and environmental protection. In addition, printed electronics enable the integration of a wide range of functional materials, such as metal nanoparticles^70–73^, carbon nanomaterials^77–79^, polymers^74–76^, or bio-inks^81^. Each printing technique offers distinct advantages for sensor fabrication, and the selection of a specific method depends on the target application and performance requirements (for an overview of key parameters, see Supplementary Table 1). SP remains the preferred method for depositing thick, viscous coatings using simple equipment, making it ideal for large-scale applications where printing resolution is not a critical factor. Its outstanding scalability and compatibility with roll-to-roll manufacturing processes make it particularly advantageous for large-scale industrial production. In contrast, IJP excels in high-resolution printing capability with minimal material waste, allowing for the precise design of small, sophisticated shapes. A key advantage of IJP is its fully digital, maskless workflow that enables rapid prototyping, in which geometries and deposition parameters can be modified in software and printed immediately without screens. With computer-aided design tools, patterns can be adjusted in a few clicks and directly transferred to the printer, shortening design cycles, reducing nonrecurring tooling costs, and enabling on-the-fly iteration for sensor arrays and gradients. The IJP is technically versatile to deal with various ink properties and chemistries, utilizing different types of droplet formation mechanisms and jetting techniques. The most popular techniques are thermal inkjet, piezoelectric inkjet, AJP, and electrohydrodynamic (EHD) jet printing^99^. The thermal IJP is praised for its simple methodology, cost-effectiveness, and suitability with water-based inks. This technique produces droplets through a nozzle by vapor bubbles produced from localized heating pulses in the ink compartment^99^. However, this method is preferred to thermally stable inks, while heat-sensitive or thermally degradable materials cannot be printed suitably. To overcome such limitations, piezoelectric IJP is widely used due to its room-temperature printing capability. This method produces a jetting droplet of ink from pressure pulses generated by a piezoelectric actuator through voltage variations^99^. The piezoelectric IJP systems offer precise droplet formation control, ensure ink stability, and are employed extensively for printed electronics and flexible device manufacturing. This method is limited in its applicability to flat surfaces due to the contact-based printing process. To tackle contactless printing on 3D or complex surfaces, more advanced type jetting, such as Aerosol IJP, is popular. This method generates aerosol from the ink by pneumatic or ultrasonic integration with sheath gas to drop ink on substrate^99^. Furthermore, the pneumatic aerosol jet is highly valued for its ability to rapidly prototype and print on or flexible substrates, thereby expanding design freedom compared to traditional inkjet methods for printing fine features (~10 µm). IJP techniques, such as EHD printing, can produce prints with high resolution in submicron to nanoscales. This technique is driven by electric field modulation to the nozzle that generates charged droplets by electrostatic forces^99^. However, it is limited by the low viscosity of the ink for better printing performance. It is increasingly favored for high-precision electronics and the fabrication of micro- and nano-sensors. Its ability to print at the nanoscale surpasses that of piezoelectric or aerosol jet methods, making it the premier choice for ultra-fine patterning. Meanwhile, 3DP further expands the design space by enabling complex three-dimensional architectures for new sensor geometries and integrated systems. This technique has wide technological provisions according to printing material, ink or feeds. These include 3DP techniques, such as fused deposition modeling (FDM), stereolithography (SLA), direct light processing (DLP), direct ink writing (DIW), and selective laser sintering (SLS), based on their operation, material capabilities, and relative advantages^100^. FDM printers print thermoplastic polymer-based filament layer by layer through extrusion from a heated nozzle. The versatile polymers for FDM printers are polylactic acid, nylon, and acrylonitrile butadiene styrene, which can also be printed with a range of nanomaterial composites. Hence, it offers a range of material possibilities with rapid prototyping, cost-effective operation, and high accessibility. It is limited by low resolution, typically in the range of a few hundred micrometers, in printed layers with inferior surface finishes due to the difficulty in extruding from narrow nozzles. For high-resolution 3D prints with micro-scale accuracy, UV-light projection-based 3DP techniques, such as SLA and DLP, are efficient^101^. These techniques utilize resin-based precursors to build 3D structures through photopolymerization with precise control using a UV laser. Furthermore, for rapid 3DP, DLP is equipped with an additional digital micromirror that advances the printing process by projecting the entire resin layer at once, rather than tracing with a laser, as in the SLA technique. These techniques offer a high resolution as low as 0.05 mm of layer height, which is better than FDM. The applicability of these methods is best suited for small parts and microfabrication, while it is only suitable for photoactive resins that restrict electronically conducting material printing. To print conducting materials or inks, the DIW method is most suitable, which extrudes viscous inks or even pastes through a fine nozzle under controlled conditions. It is enabling direct writing of functional inks, biomaterials, or nanomaterials with extrusion control by pneumatic, screw or piston-based functions. It excels at creating complex, multi-material, and large-scale 3D structures. The method’s flexibility in inks makes it suitable for printed electronics and tissue engineering, albeit with lower resolution than resin-based techniques^102^. To achieve complex geometry with high mechanical strength, SLS 3DP is preferred. It utilizes a high-power laser to selectively sinter powdered materials (e.g., metal powders, nylon, polyamides) without the need for support structures. It is widely used in aerospace, automotive, and dental industries for functional prototyping and end-use parts. Its main drawback is the high cost and slow build times^101^. DLW complements these approaches by enabling solvent-free, additive-free fabrication of conductive microelectrode patterns through the photothermal conversion of carbon-rich precursors (e.g., polyimide) into laser-induced graphene. This technique provides exceptional resolution and design freedom for producing porous, doped, or hierarchical microstructures in a single step. AJP extends this portfolio by enabling the deposition of fine, continuous lines with feature sizes down to several micrometers and excellent conformity on nonplanar or flexible substrates. Unlike IJP, which relies on direct droplet ejection, AJP employs an aerodynamic focusing mechanism to deliver a narrow aerosolized stream of ink toward the substrate. This process provides exceptional versatility in printing on irregular or curved surfaces, including textiles, polymers, and even biological tissues, such as leaves. Furthermore, AJP accommodates a broad range of ink viscosities, bridging the gap between the low-viscosity formulations used in IJP and the thicker pastes required for SP, while maintaining high resolution and reproducibility. In all five techniques, the resulting device performance is determined by the interplay between ink composition, printing resolution, substrate adhesion, and post-print processing. SP is recommended for large-area electrochemical electrodes and low-cost arrays (e.g., ion, pH, and ROS) on flexible films where thick, robust conductors are needed. IJP is practical for fabricating high-resolution, conformal electrodes and biofunctional layers with minimal ink usage, while AJP is preferred for direct on-leaf or curved-surface features requiring fine lines and good conformity. DLW is advantageous for porous, binder-free carbon microelectrodes and chemiresistive, humidity or strain sensing, and 3DP is best for structural elements like microneedle patches, housings, and clips, that are subsequently functionalized by SP/IJP/AJP. In summary, printing technologies offer an additive, adaptable, and environmentally sustainable approach to sensor development that enables rapid prototyping, minimal waste, and extensive design flexibility.

### Screen printing for crop sensor development

**Fig. 2:**
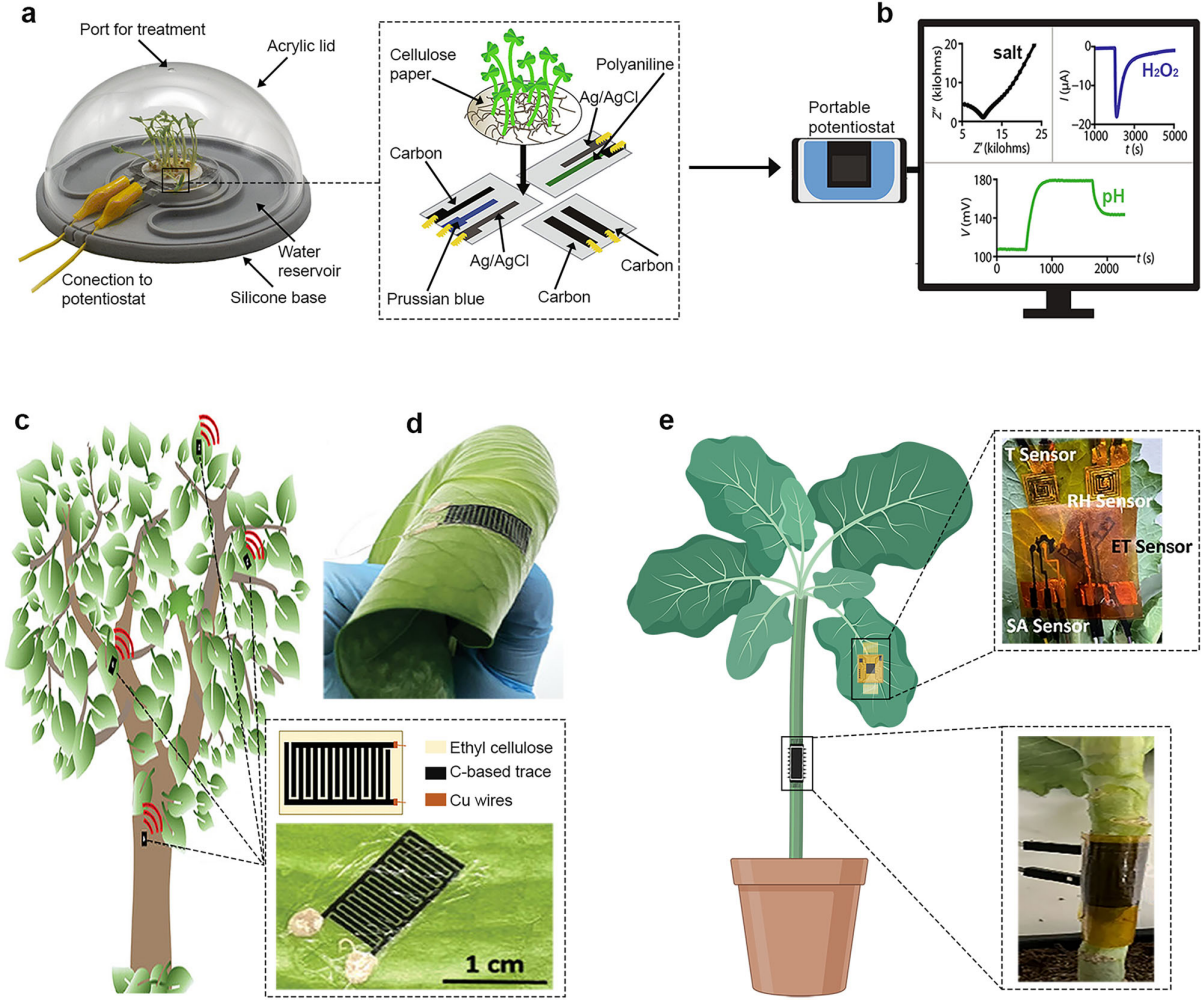
Screen printing (SP) sensors for crop monitoring. **a** Schematic of TETRIS, showing sensor fabrication, growth of seedlings, and recording of measurements using a standard laboratory potentiostat. **b** Simultaneous measurement of ion concentrations, H_2_O_2_ levels, and pH variations enables comprehensive monitoring of dynamic chemical changes. **c** Illustration of leaf surface moisture detection at four different locations in the tree canopy. **d** Sensor flexibility. **e** Photos of the plant-mounted sensors on the stem and leaf. Parts **a**, **b** are adapted from Coatsworth, P. et al.^14^, licensed under CC-BY 4.0 (https://creativecommons.org/licenses/by/4.0/). Parts **c**, **d** are adapted from Strand, E.J. et al.^53^, licensed under CC-BY 4.0 (https://creativecommons.org/licenses/by/4.0/). Part **e** adapted from Hossain, N.I. et al.^118^, licensed under CC-BY 4.0 (https://creativecommons.org/licenses/by/4.0/).

SP is a versatile, centuries-old technique that has been successfully adapted for modern materials science, with applications ranging from energy,^103–105^ via electronics^106–108^ to sensing^109–111^. Its primary advantages are simplicity and scalability (massively used in industrial series production), enabling the rapid deposition of commonly 5–1000 µm thick layers^112^ over large areas at a low cost^113^. SP has recently emerged as a versatile technique for fabricating diverse plant sensors, including screen-printed carbon electrodes^14,53,114^, wireless^115,116^, and wearable^117–119^ devices that can be inserted into soil or attached directly to plants for real-time monitoring of their physiology and environmental conditions. Among them, a time-resolved electrochemical technology for plant root environment in situ chemical sensing (TETRIS^14^) has been developed to enable continuous chemical monitoring using low-cost SP electrochemical sensors (Fig. 2a). The platform integrated three screen-printed sensors on polyester, including a potentiometric pH sensor composed of screen-printed carbon and Ag/AgCl electrodes where polyaniline was electropolymerized on the carbon surface to form a pH-responsive working electrode, an amperometric hydrogen peroxide (H_2_O_2_) sensor consisting of Prussian blue-mediated carbon, carbon, and Ag/AgCl electrodes with a PB-carbon working electrode, and an impedance-based salt sensor with two carbon electrodes for ion monitoring (Fig. 2b). Results demonstrated species-dependent (kale, tomato, and rice) and ion-specific (nutrients and heavy metal ions) uptake behaviors, influenced by variables, such as plant development stage, ion uptake identity, and channel modulation. In addition, the ML model trained on physicochemical descriptors successfully predicted normalized uptake rates, emphasizing the system’s potential for data-driven crop screening. RH at the plant-environment interface is a key indicator of plant health, as it controls physiological functions like transpiration, nutrient transport, and temperature regulation. In addition, elevated RH, especially under favorable temperatures, can promote stomatal opening and microbial colonization, increasing susceptibility to pathogens and affecting irrigation and crop protection strategies. To this end, a fully SP, ultra-thin (6 µm) capacitive sensing platform has been designed for direct and noninvasive RH monitoring directly on living plant tissues^53^. The device uses a sandwich capacitor architecture in which carbon-based electrodes are encapsulated between two layers of ethyl cellulose and applied via a temporary tattoo method (Fig. 2c). This design allows the sensor to be stretchable (Fig. 2d), thus allowing flexible adhesion to different plant surfaces while achieving a record-high sensitivity of up to 1000 pF/%RH. Monitoring of RH using electrochemical impedance spectroscopy (EIS) demonstrated reliable multi-day operation with high accuracy compared to commercial devices. The response time, however, was slower because of the diffusion dynamics of material encapsulation. Further advances in encapsulation throughput and device miniaturization could reduce this delay and increase suitability for precision agriculture deployments.

Since salicylic acid and ethylene are key signaling molecules primarily involved in pathogen defense and general stress responses, including senescence, real-time monitoring is essential for understanding crop health. In order to monitor these critical parameters continuously, noninvasively and multiplexed, a fully integrated, Internet of Things (IoT)-enabled sensor has been developed capable of wireless real-time data transfer to remote devices for centralized analysis and decision-making^118^. The low-cost, flexible electrochemical sensors were calibrated using laboratory protocols and verified during 60-day field experiments on pepper, cabbage, and tomato plants (see Fig. 2e) exposed to variable water stress^118^. The resulting data revealed dynamic correlations between phytohormone levels and water transport. This work bridges a long-standing technological gap by providing minimally invasive monitoring with spatiotemporal resolution, unlike traditional methods, such as high-performance liquid chromatography or gas chromatography-mass spectrometry, which are destructive, time-consuming, and lack temporal resolution. While continuous monitoring of physiological and chemical parameters, stress-related markers, is crucial for assessing plant status and environmental responses, certain agricultural scenarios demand specific detection of pathogenic agents. In such cases, molecular recognition of viral or microbial targets becomes essential, particularly for early-stage diagnosis and intervention^120^. Advances in SP platforms have extended their application to more complex biosensing configurations. One illustrative case involves carbon SPEs modified with electrodeposited gold nanoparticles (AuNPs), enabling the covalent immobilization of thiolated DNA probes for the label-free electrochemical detection of citrus tristeza virus (CTV). This system demonstrated high selectivity and sensitivity through EIS, with successful validation in spiked plant samples^121^. Expanding on this approach, a complementary study integrated solid-phase isothermal recombinase polymerase amplification directly on AuNP-modified SPEs, achieving in situ nucleic acid amplification and detection of CTV at room temperature, without thermal cycling or labeling. The resulting sensor reached a limit of detection of 1 pg µL^−1^ with high reproducibility, offering a viable strategy for portable and field-deployable diagnostics^122^. Together, these examples underscore the versatility of SP technologies for integrating molecular recognition and amplification within low-cost, scalable sensing platforms tailored for in situ agricultural pathogen monitoring at the relevant care point places and times in the field.

Despite all the achievements and advances, SP still faces several technical challenges that must be addressed. Ink composition and rheology are among the most critical factors, as optimizing these properties is essential for achieving reliable performance and high-resolution patterning. SP requires inks with high dynamic viscosity, typically in the sup range of 1000–10,000 mPa s, to prevent bleeding (unwanted ink spreading) and ensure uniform transfer across the screen stencil^85^. Formulating printable viscous inks that retain both stability and functionality (e.g., sensitivity and selectivity of the final sensor) is a major challenge and has become an increasing focus for many materials industries^123–126^. While the development of advanced inks based on low-dimensional materials is very promising and has made remarkable progress^84,87,127^, their strong tendency to aggregate at high viscosity poses major difficulties in achieving a stable ink. In practice, the ink composition must balance multiple requirements beyond viscosity, including conductivity, sufficient adhesion to the substrate, curing properties, and effective functionalization. However, achieving all these ink features requires complex mixtures of active material with solvents, binders, and additives that can significantly influence the functionality of the final ink (e.g., limiting access to functional groups, thereby reducing selectivity)^128^.

Resolution limitations are also important, as unlike photolithography or IJP, traditional SP exhibits coarser resolution (generally tens to hundreds of micrometers) due to inherent constraints including ink spreading, screen mesh geometry, and stencil definition^85^. Although this resolution is sufficient for many applications, it remains inadequate for the new generation of fine microelectronic devices^129^. These micro-technologies play a pivotal role in sensor development, as the emphasis is put on sensor size (plants and their organs can be smaller than a centimeter), reducing the material load and, thus, the ecological footprint. While the broad compatibility of SP technology with diverse substrates is considered a major advantage, multiple challenges persist. Specifically for plant sensors, the surface of living plant organs is irregular and dynamic, and therefore, direct printing on leaves or stems via mesh and stencil templates is unfeasible. Moreover, the curing or sintering of SP inks typically requires elevated temperatures, posing an additional limitation since plants are living organisms. Another key limitation of SP technology is the high material consumption, as a significant volume of ink is required to cover the entire stencil mask. In addition, SP suffers from considerable irreversible ink loss during electrode fabrication, mainly due to retention on the mesh, stencil, and squeegee, resulting in significant material waste^85^. This limitation becomes especially challenging when using high-value biomaterials (crucial for selectivity) such as aptamers, antibodies, or DNA probes, which are expensive to produce and typically available in limited quantities. As a result, using SP for these bio-inks is neither economically nor practically suitable, leading to the functionalization of sensors predominantly via the drop-casting method. Although straightforward, this additional step still requires relatively large material volumes and suffers from limited reproducibility^130^, a major bottleneck in sensor development^131^.

As ink properties, particularly viscosity and colloidal stability, are critical for proper SP, further optimization in this area will be increasingly important. For instance, stable and highly viscous ink is essential for fabricating wearable plant patches^53^ (see Fig. 2c), enabling direct monitoring of trace-level analytes at the leaf interface. A promising direction involves the development of smart inks based on low-dimensional materials and conductive additives, which offer high electrical conductivity, mechanical flexibility, and tunable functionality, thereby enhancing sensor performance and expanding their application profile. Particular attention will shift to environmentally friendly, highly concentrated water-based inks that could progressively replace conventional organic solvent-based formulations. Although organic solvent-based inks remain widely used due to advantages, such as controlled drying, optimized rheology, and sufficient adhesion, the use of toxic solvents (glycol ether acetates, cyclohexanone, and other ketone-based solvents)^132,133^ limits their deployment in sustainable applications. In this respect, the alternative organic solvents or water-based formulations are particularly beneficial for printing on biodegradable substrates^134^. Another key objective is improving print resolution and accuracy by enhancing screen and stencil technologies, such as finer mesh geometries or photopatterned stencils, enabling micrometer-scale features. This improvement would allow miniaturization, thus the integration of robust sensor arrays into compact environments, such as greenhouses or hydroponic systems, where space is limited. In summary, ongoing research and innovations ensure that SP will be the cornerstone of printed electronics, providing a cost-effective, versatile, and readily scalable technique, particularly in scenarios where miniaturization and ultra-high resolution are not essential requirements.

### Inkjet printing for crop sensor development

IJP remains one of the most cost-efficient techniques for device prototyping and manufacturing^87^. This technique offers considerable freedom of digital design, noncontact, and additive fabrication by depositing picolitre-scale droplets, enabling the formation of precise and high-resolution patterns^112,135^. Initially developed for graphic applications, IJP technology has become an attractive deposition technique for materials science and, similar to SP, has gained significant attention from the scientific community, covering applications from sensors^136–138^ and energy^139–141^ to biotechnology^142–144^. In contrast to conventional SP, this method eliminates the need for masks or meshes, enabling direct transfer of patterns from digital designs to substrates^145,146^. Moreover, inks for IJP can be formulated as water-based systems, a feature that is difficult to achieve in SP due to viscosity and wetting constraints^87,147,148^. IJP significantly reduces material consumption (on the order of micrograms^148,149^) by precise deposition and negligible material losses, thus significantly reducing costs. Consequently, it could enable printing of expensive biological materials (e.g., aptamers, antibodies, DNA probes) which are not economically viable with SP due to the large material volume, the large material requirements and the substantial waste generated. Compared to SP or photolithography, IJP is particularly attractive due to its low material consumption, high-resolution, and printability on nontraditional substrates, such as irregular natural surfaces like plant leaves^97,98^, all without the need for sophisticated cleanroom facilities required by conventional lithographic methods.

**Fig. 3:**
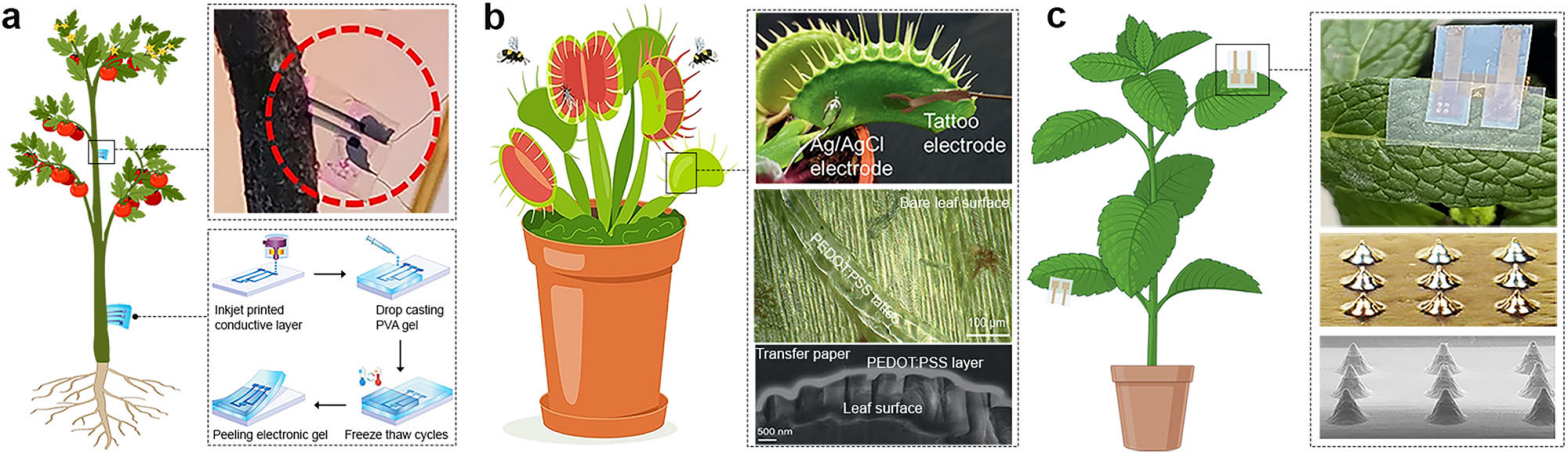
Inkjet printing (IJP) sensors for crop monitoring. **a** Illustration of the sensors mounted on the plant stem and schematic of the fabrication steps of the electronic cryogels. **b** Illustration and photos of the tattoo electrode on *D. muscipula* leaf and SEM image of the leaf surface covered with the electrode. **c** Photos of the sensor attached to the mint leaf with 100 µm high microneedles and SEM pictures of the 3 × 3 microneedles. Part **a** is adapted from Bihar, E. et al.^151^, licensed under CC-BY 4.0 (https://creativecommons.org/licenses/by/4.0/). Part **b** is adapted from Meder, F. et al.^97^, licensed under CC-BY 4.0 (https://creativecommons.org/licenses/by/4.0/). Part **c** is adapted from Rosati, G. et al.^150^, licensed under CC-BY 4.0 (https://creativecommons.org/licenses/by/4.0/).

Like SP, IJP technology has become increasingly prominent as a method for developing sensors for plant physiological monitoring^54,97,150–152^. For example, IJP has been used to deposit highly conductive PEDOT:PSS ink directly onto the biocompatible polyvinyl alcohol cryogels. Unlike SP, which often requires high viscosity inks and leads to thicker layers, IJP offered excellent resolution, but especially compatibility with an unconventional soft, hydrated substrate. The sensors were fabricated by IJP PEDOT:PSS traces on glass through three passes, followed by drying at 90 °C for 10 min, coating with a PVA hydrogel, peeling and re-encapsulation with a second PVA layer, and freeze-thaw crosslinking at −20 °C to produce a freestanding electronic cryogel implant (Fig. 3a). This approach allowed for monitoring plant ion fluxes and hydration status in tomato stems, which is essential for understanding nutrient uptake, water transport, and plant responses to stress^151^. Further use of IJP enabled the design of tattoo-like sensors for noninvasive, long-term measurement and stimulation of electrophysiological signals in plants, which are crucial for advancing our understanding of plant signaling. In this context, ultra-conformal PEDOT:PSS-based electrodes have been developed to address key issues in plant interfacing, including invasiveness, low mechanical stability and short operational lifetime. Manufactured on tattoo transfer paper and incorporating flexible silver interconnects, this ultra-thin device (<3 μm) adheres to leaf surfaces via van der Waals forces, requiring no binders or electrolyte gels (Fig. 3b). Such a sophisticated approach enabled the recording of electrophysiological signals in three plant species (*Dionaea muscipula, Arabidopsis thaliana*, and *Codariocalyx motorius*). The same electrodes were also used to trigger trap closure in *Dionaea muscipula* by electrical stimulation, demonstrating bidirectional functionality. Signal acquisition remained reliable for over 10 days, with simple rehydration of the tattoo layer restoring performance, thus highlighting the system’s robustness^97^. Even IJP can be utilized for the fabrication of microneedle architectures (mainly produced via 3DP, see “Three-dimensional printing for crop sensor development”), offering a scalable and maskless approach for developing minimally invasive sensing interfaces. Researchers developed a method for the direct printing of conductive microneedles using silver nanoparticle inks and a modified drop-on-demand printing setup equipped with localized heating. This configuration enabled rapid in situ solvent evaporation, allowing for the vertical growth of mechanically robust and electrically conductive microneedles without post-processing (Fig. 3c). The printed microneedles were used for in vivo EIS on mint leaves as a promising technology to monitor plant nutrient status^150^.

Although IJP offers advantages over conventional methods, it faces significant technical limitations that hinder its broader use in large-scale sensor development. These issues relate to scalability^112^ (compared to high-throughput SP) and primarily to ink composition, with the critical challenge being the preparation of submicron particles (<500 nm) that can pass through the narrow nozzles of the inkjet printhead^112^. Additional concerns include substrate compatibility^112^ and, above all, the limited availability of refillable inkjet material printers, which are absent from commercial suppliers. Creating stable, printable inks presents a major challenge, as inks must exhibit precisely controlled viscosity (1‒20 mPa s), surface tension, and the required submicron particle size^145,153^. Another critical bottleneck related to ink composition is printhead clogging, often caused by fast-evaporating inks or particulate deposits in the nozzle, which leads to irregular droplet ejection and degraded print quality^145,153^. Although self-cleaning mechanisms help mitigate these issues, ensuring stable performance during extended print sessions remains difficult. Additionally, metal-based inks can corrode nozzles or form deposits, further reducing print quality^153^. While strategies, such as anti-fouling coatings and ink recirculation systems, are under investigation, regular nozzle maintenance remains an unavoidable and laborious aspect of IJP^112^. One of the major limitations of IJP compared to SP is its poor compatibility with various substrates, particularly glass and metals. Properties, such as different hydrophobicity, porosity, and thermal stability of the surface, significantly affect droplet behavior and adhesion^154^. Unlike SP and lithography, which rely on physical stencils or meshes and require post-processing, IJP is a digital method in which designs can be modified with a few computer clicks, eliminating the need for the creation of new masks. This enables rapid prototyping and the precise, direct printing of functional materials onto irregular, dynamic surfaces, such as plant leaves and stems^97,98^. In conclusion, IJP technology still faces significant challenges despite its considerable advantages. Advances in this technology require addressing ink formulation, improving substrate compatibility, and, most importantly, an expanded portfolio of printable functional materials.

Future improvements in printing techniques must be aligned with the specific requirements of agricultural sensing, including robustness under variable environmental conditions, low-cost fabrication for dense deployment, flexibility for conformal integration on leaves and stems, and biodegradability to minimize field waste. Advancements in printing resolution, material compatibility, and substrate engineering can directly translate into more durable, sensitive, and field-ready sensors for precision agriculture. IJP technologies are rapidly evolving due to advances in materials science, engineering, and digital manufacturing techniques. Future advancements are expected to enhance high-resolution printing through cutting-edge techniques, such as EHD printing, also known as E-jet. Unlike conventional inkjet methods, EHD printing employs an electric field to generate ultra-fine droplets, achieving resolutions down to a few hundred nanometers^155,156^, thus achieving even greater miniaturization and saving both materials and cost. This ultra-high resolution could allow printing of multiplexed electrochemical sensors on small spaces (leaves, stems, fruits), enabling simultaneous monitoring of different phytohormones or nutrient ions with minimal material consumption. As with SP, significant progress will depend on the development of functional inks, as the limited availability of printable smart inks remains a major barrier, mainly due to the difficulty of achieving small particle sizes. Innovative formulations based on low-dimensional materials (e.g., graphene derivatives^127^, MXenes^84^, and TMDs^87^) are promising to address key challenges in this field. Currently, the availability and cost of high-precision inkjet and EHD printers pose significant practical barriers, shaping the current technological baseline for future innovation. The most widely used systems, including the Fujifilm Dimatix and SUSS MicroTec LP50, employ industrial-grade printheads, such as Samba, Konica Minolta, Canon, and Xaar that provide excellent resolution but remain bulky and expensive (Dimatix ≈ USD 70,000 and ≈ USD 250 per cartridge). These platforms illustrate the existing capabilities from which next-generation portable, miniaturized, and field-deployable printing systems are expected to evolve. Their continued development toward lower-cost, compact architectures will be crucial for translating high-resolution inkjet and EHD printing into real-world agricultural applications. In parallel, a growing number of studies have successfully repurposed commercial desktop inkjet printers as ultra-low-cost platforms for fabricating electrochemical sensors. For example, consumer-grade systems have been adapted for the deposition of nanomaterial-based inks on flexible substrates, such as Mitsubishi paper mills NB-TP-3GU100, enabling the prototyping of functional electrodes at a fraction of the cost of industrial equipment. Among them, models like the Epson XP-15000 (ca. USD 400) have been employed for printing metallic nanoparticle inks, particularly silver and gold, underscoring their potential to democratize the fabrication of conductive patterns and electrochemical interfaces^157,158^. Although these printers lack fine droplet control and resolution, their accessibility and ease of use make them attractive for educational settings, rapid testing, and sensor deployment in low-resource environments. A more forward-looking perspective envisions the development of compact, portable printers, analogous to those in photography, which allow users to print high-resolution photographs immediately after the photo is taken. For example, such portable printers could be mounted on drones (already existing with 3DP^159^) to directly print sensors onto leaves, fruits, or plant-attached patches in big fields, and thus have the potential to revolutionize on-site material printing and sensor deployment. Overall, the evolution of IJP technology will be shaped by higher resolution, next-generation ink formulations, seamless integration into scalable and robust production processes, and, most importantly, the miniaturization and enhanced portability of the printers.

### Three-dimensional printing for crop sensor development

**Fig. 4:**
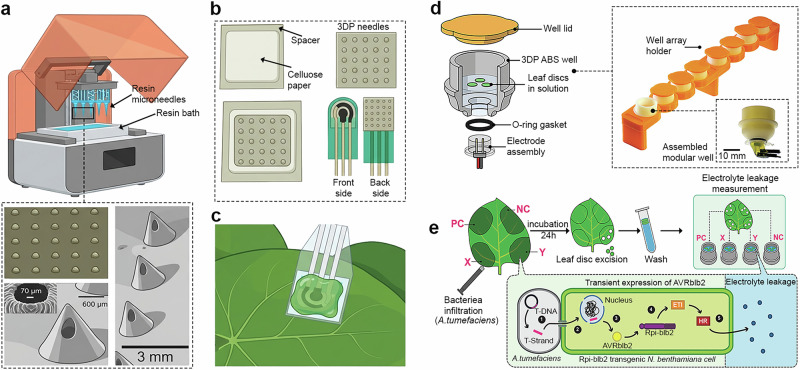
3D printing (3DP) sensors for crop monitoring. **a** Microneedle fabrication via resin filament and its design. **b** Sensor construction. **c** Sensor directly attached to the leaf via the needle properties. **d** Schematic of individual measurement well assembly during measurement and the photograph of the 3DP well assembly. **e** Schematic of the experimental procedure for leaf disc assay in *Nicotiana benthamiana*. PC and NC denote positive and negative control, respectively. PC is an AVRblb2-carrying *Agrobacterium tumefaciens* suspension of OD600 = 0.1, and NC is an empty vector (EV)-carrying *A. tumefaciens* suspension of OD600 = 0.1. X and Y contain discrete dilutions of AVRblb2-carrying *A. tumefaciens*, varied between experiments. Inset: simplified molecular schematic of HR generation via agroinfiltration. Parts **a**–**c** were created in BioRender. Panacek, D. (2026) https://BioRender.com/bub5cta. Parts **d**, **e** are adapted from Collins, A.S.P. et al.^39^, licensed under CC-BY 4.0 (https://creativecommons.org/licenses/by/4.0/).

3DP has evolved into a powerful platform for materials science^160–163^. Unlike subtractive methods (CNC machining, laser cutting, and milling), 3DP creates objects layer by layer from digital designs, allowing unprecedented freedom in material shape, structure and composition^164^. In the last decade, 3DP, which was initially used primarily for rapid prototyping of plastic parts, has expanded into diverse applications, including printing of tissues^165–167^, energy-related systems^168–170^, and flexible electronics^171–173^. Its appeal stems from its ability to accommodate complex geometries without extensive tooling, minimize material waste and integrate multiple materials within a single design. These advantages have positioned 3DP as an enabling technology across sectors ranging from aerospace^174,175^ and biomedical engineering^176,177^ to smart sensors^178,179^. This printing method is more recent than SP and IJP but has nevertheless attracted considerable interest in developing sensors for plant monitoring^39,40,180,181^. Since 3DP faces a significant limitation in the low functionality of the materials contained in the filaments, it is difficult to directly fabricate the working electrodes (confining the recognition element) using 3DP alone. 3DP is therefore used mainly as a complementary approach alongside techniques, such as SP or IJP. For instance, 3DP, namely masked-stereolithography apparatus (MSLA), was used for the large-scale fabrication of microneedles mounted to the SP sensor platform (Fig. 4a, b) and thus enabled direct monitoring of physiological processes from the plant leaf (Fig. 4c)^40^. Although it has been demonstrated that microneedles can be created by IJP^150^ (see Fig. 3c), the usage of 3DP allowed advanced design by fabrication of hollow microneedles (HMAs), which were capable of minimally invasive sampling of apoplastic fluids (liquid present in the extracellular spaces of plant tissues, involved in nutrient transport and stress signaling). The method enabled rapid prototyping of microneedles with sharp tips (<30 μm) and integrated hollow channels, achieving highly reproducible structures using a cheap (ca. USD 450) MSLA 3D printer. Mechanical tests demonstrated that HMAs could reliably penetrate plant tissues without significant deformation. In addition, in vitro and in vivo tests confirmed their ability to extract sufficient fluid volumes (ca. 15 µL) for proper electrochemical analysis. By combining these sophisticated microneedle patches with SP electrodes, the sensor platform was capable of real-time detection of key biomarkers related to plant stress responses, such as H_2_O_2_, glucose, and pH across different plant species. This approach offers notable advantages, including low manufacturing cost (<USD 1/device) and high versatility in analyte detection. However, the fluid-based extraction strategy is highly dependent on the plant species, as successful electrolyte extraction requires a sufficient volume of electrolyte (apoplastic fluid) in the leaf. Therefore, this method cannot be used on unsuitable plants, such as plants with thinner or drier leaves. To enable high-throughput, cost-effective, and parallel monitoring of programmed cell death in plant tissue, a 3DP sensing device has been developed for continuous impedance-based quantification of electrolyte leakage^39^. The methodology capitalized on 3DP to fabricate modular, reusable measurement wells tailored for optimal sensor placement and minimal sample volumes, thereby enhancing sensitivity and reducing operational complexity (Fig. 4d). The experimental workflow involved controlled infiltration of plant leaves with a bacterial suspension using a syringe, excision of leaf discs after incubation, and their transfer into measurement wells for immediate, continuous conductivity monitoring, enabling rapid detection of microscopic cellular damage (Fig. 4e). Quantitative readouts were achieved by analysing solution conductivity from bacteria-infiltrated leaf discs, capturing both macroscopic and microscopic stages of cell death within hours. The system’s architecture allowed frequency-resolved EIS across eight channels simultaneously, drastically accelerating data acquisition compared to traditional methods. Conventional assays typically rely on manual electrolyte leakage measurements at sparse time intervals, requiring labor-intensive sampling and offering limited throughput without real-time monitoring capability.

Despite rapid progress, several technical and practical challenges hinder the deployment of 3DP in sensor development and broader materials research. One of the primary challenges lies in the diversity and functionality of materials. Although the library of 3DP materials is expanding, it remains relatively narrow and often tailored to specific applications. Advanced materials for aerospace^182^, automotive^183^, or electronics^184^ typically demand specialized printers and processes. These high-end systems (e.g., metal laser sintering) are costly and require expensive precursors, limiting accessibility. Consequently, identifying materials that fulfill the mechanical and chemical criteria for plant sensing remains a major obstacle. In addition, conventional polymers (e.g., PLA, ABS, and epoxy resins) often exhibit low or no conductivity and flexibility^185^. Another major limitation is the lack of functionality (chemical groups on the surface) in printable materials^186^. Since most materials are based on plastics, resins, or carbons with inert surfaces, they fail to provide the chemical specificity required for sensing^187^. Similarly to SP, 3DP has a significant disadvantage in the material consumption to produce the filament, which is impractical when using expensive materials such as high-cost metals or biological compounds^188^. Additionally, 3DP has a relatively low resolution, as most techniques cannot match the precision of traditional microfabrication or machining. Feature sizes below several tens of micrometers are a practical limit for conventional 3D printers, making it challenging to achieve nanoscale geometries or mirror-smooth surfaces^189^. Improving the resolution of printers without loss of speed is an active area of technical development^190,191^. Scalability, particularly the slow printing speed, is another issue, as fabricating a single object can take hours, while producing larger or multiple components can take days^192^. As a result, 3DP is primarily used for prototyping, small-batch fabrication, or custom one-off designs. Solving these issues is a primary focus of ongoing materials science and engineering research. Innovations are required in printer hardware (to improve resolution and speed), printing materials (to expand the palette and increase performance) and process engineering (to ensure uniform quality and scalability). Overcoming these limitations will be key to fully exploiting the potential of 3DP in industry and scientific research.

Future 3D printers are anticipated to process an increasingly diverse range of materials, including not only conventional plastics, metals and ceramics but also functionalized^193,194^, biological^195,196^ and even living materials^197,198^. Research is also advancing toward stimulus-responsive materials for 4D printing that can change shape or properties over time, adding a dynamic dimension to printed structures^199,200^. This can be used in developing smart sensors used in agriculture to monitor changing weather conditions^201^. Furthermore, new additive techniques are pushing the limits of resolution to the microscale and beyond. For example, two-photon polymerization (laser-based 3DP) can produce structures with sub-micron features and functions almost as a 3D printer for nanostructures^202–204^. As these high-resolution printers are further developed and adapted for larger-scale use, they could enable the fabrication of tiny, precise components for microfluidics, electronics, and sensors. In plant monitoring, high-resolution printing could support the production of microsensors or nano-device systems that interface with plant cells or tissues in a minimally disruptive manner, significantly expanding the toolbox for investigating plant biology. For 3DP to take off, future systems must print faster and greener. One direction is the development of printers that operate in parallel, enabling the printing of multiple materials simultaneously. For instance, the system can be equipped with multiple synchronized print heads, which pave the way for producing entire electronic devices or sensors in a single print^205–207^. Such approaches could significantly accelerate production and enable the efficient fabrication of high-volume batches or structurally large components. Another critical aspect is sustainability, with research focusing on recyclable and biodegradable printing materials derived from biomass or recycled printing waste to produce new filaments^208–211^. In agriculture, this could manifest as biodegradable 3D-printed sensors that remain in the field post-use and naturally decompose, eliminating waste and reducing the environmental footprint. 3DP in materials science rapidly evolves from a prototyping tool into a core manufacturing and research technology. The future of 3DP in sensor development is expected to converge with complementary techniques, such as SP or IJP, enabling the production of integrated devices that combine structural versatility with functional precision. For instance, inkjet-printed nanoscale sensing components can be seamlessly incorporated into 3D printed platforms containing energy storage units, such as micro-supercapacitors or fluidic microchannels. This hybrid approach offers unprecedented design freedom and allows multifunctional sensor systems to be fabricated as unified, compact architectures adapted for device integration and field deployment. By addressing current limitations and leveraging emerging innovations, 3DP is poised to redefine the design and deployment of functional materials for next-generation electronic platforms.

### Direct laser writing-based hybrid printing for crop sensor development

DLW is a powerful technique for micro- and nanoscale patterning of graphene or carbon-based layers for sensing electrodes^59,212^. It has been widely employed to fabricate electronically conductive patterns on substrates, such as polyimide (PI) and other natural or synthetic carbon-based polymers, through direct laser-induced conversion of polymer into graphene or carbon^213^. Unlike cutting or MSLA-based 3DP methods, DLW produces a thick, surface-engraved graphene or carbon layer using pulsed or continuous lasers in ambient conditions and is regarded as a partially destructive approach^214^. DLW enables rapid, solvent-free fabrication of microelectrodes in a single step, without additives or binders, and allows precise patterning across diverse geometries^215^. During laser–material interaction, key parameters, such as layer thickness, porosity, and in situ doping, can be finely tuned, offering tailored electrochemical properties^216^. Owing to these advantages, DLW has been applied in the fabrication of flexible micro-supercapacitors and micro-battery electrodes with high storage capacities^60^. For sensing applications, DLW is often combined with other techniques to pre-deposit dopants or post-deposit sensing layers onto patterned surfaces. It has also been integrated with film-transfer methods to produce flexible and stretchable strain sensors, expanding its utility in wearable electronics. Given the demand for lightweight, conductive, and flexible patterned electrodes, DLW has enabled effective strategies for fabricating plant monitoring sensors targeting RH, temperature, VOCs, and mechanical strain^217–219^.

**Fig. 5:**
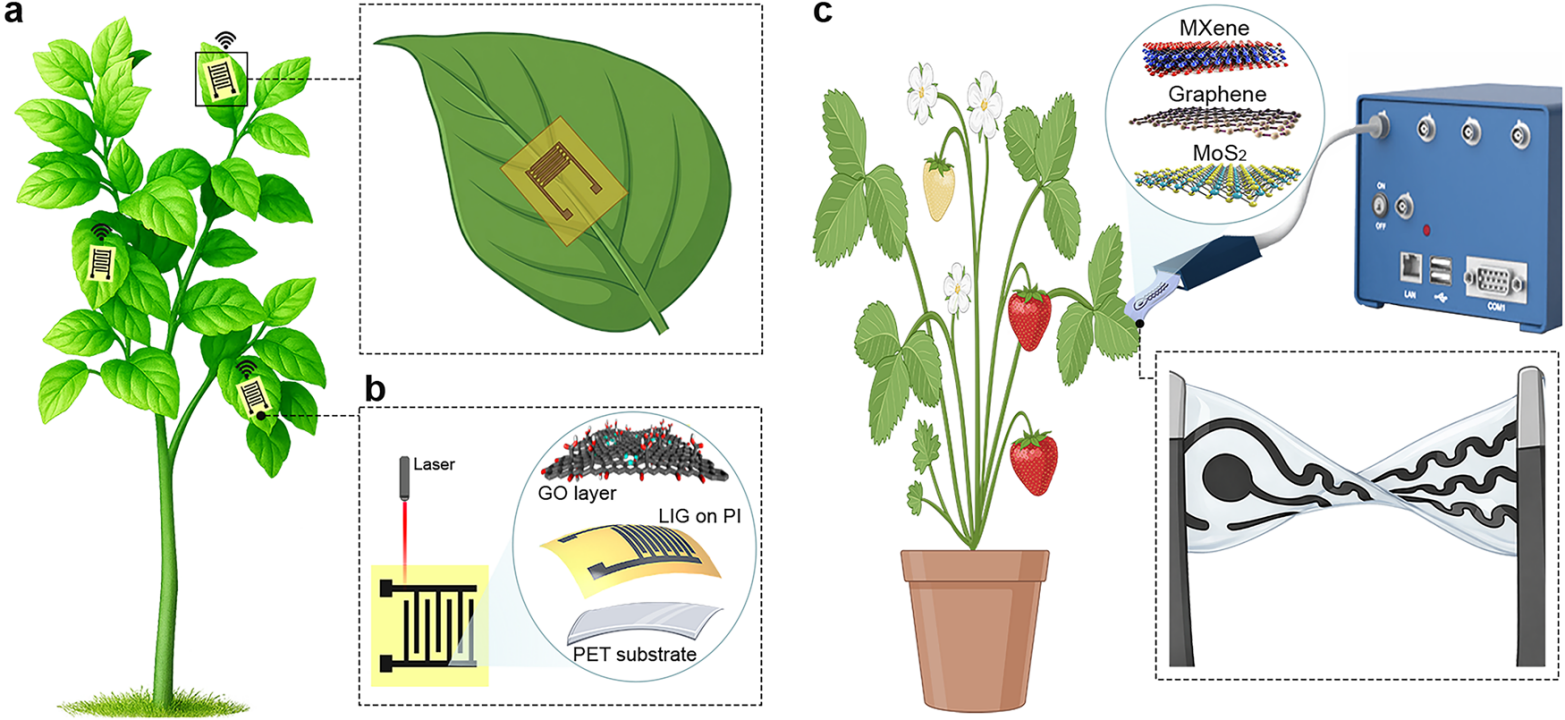
Direct laser writing (DLW)-based sensors for crop monitoring. **a** Setup and design of the LIG sensor mounted on tree leaves. **b** Demonstration of the DLW method for the fabrication of LIG. **c** Setup of the measurement and design of the LIG functionalized with MXene and MoS_2_.

For instance, DLW has been employed to fabricate LIG and graphene oxide (GO)-based humidity sensors for real-time monitoring of plant transpiration and plant health^16^. This approach allows a noninvasive and wide distribution of sensors over a large part of the tree, enabling robust and time-dependent monitoring of leaf water content (Fig. 5a). In this procedure, a PI substrate serves as the carbon source for patterning interdigitated LIG, while a GO film is cast onto the structure as a capacitive sensing layer (Fig. 5b). Although GO functions as the humidity-sensitive material, LIG plays a critical role by modulating permittivity across humidity levels. This sensor, due to its flexibility, can be mounted directly on a leaf to detect noncontact humidity changes associated with stomatal behavior through GO–water interactions. Real-time water content sensing demonstrates a rapid capacitive response during water supply and its subsequent decline. DLW also enables the fabrication of LIG-based resistive sensors for temperature, strain, and light intensity, expanding its utility for plant and environmental monitoring^217^. DLW parameters, including laser power and patterning style, strongly influence electrode morphology and humidity sensing performance in plant-based applications^218^. Beyond PI, natural biopolymers, such as TEMPO-oxidized cellulose, have also been used to produce moisture-stable electrodes via DLW for sensing plant water status^219^. Precise micro-patterning is essential for developing electrochemical sensors that detect plant chemical biomarkers, particularly when multiple electrode arrays are integrated on a single substrate, an area where DLW excels. Such sensors can be fabricated by printing LIG directly on PI, or transferred onto stretchable substrates, such as polydimethylsiloxane (PDMS) for flexible sensing platforms^220,221^.

Another example of DLW is a flexible electrochemical sensor designed for on-leaf detection, which combines LIG, MXene, and MoS_2_ to detect gallic acid, a phenolic compound that is accumulated in plants as an antioxidant in response to stress conditions, such as salinity, drought, or pathogen exposure^222^. Serpentine tri-electrodes were fabricated by laser writing porous LIG on PI tape, followed by spin coating with PDMS and curing at 120 °C for 10 h before delamination to obtain a stretchable LIG/PDMS substrate. The central trace was insulated with PDMS, the reference electrode was patterned with Ag/AgCl ink, and the working electrode was sequentially drop-cast with MoS_2_ and Ti_3_C_2_ MXene before air drying. Due to its compact size and mechanical flexibility, the sensor adheres directly to leaves and enables in situ, real-time monitoring (Fig. 5c). The LIG three-electrode system was patterned onto PI and transferred onto a PDMS substrate via resin casting, enabling highly flexible functionality. To enhance sensitivity and selectivity, the working electrode was functionalized with the two-dimensional materials MXene and MoS_2_, which facilitated voltametric detection of gallic acid through a distinct redox response. Under salt-induced stress, it delivered rapid and precise electrochemical signals corresponding to the dynamics of gallic acid content in leaves, demonstrating its potential for continuous, noninvasive monitoring of plant stress physiological response.

DLW offers distinct advantages over techniques, such as SP, IJP, and 3DP, owing to its single-step, binder-free process and high-resolution scalability. However, it remains underexplored in the sensor field and presents several technical and fundamental challenges. A primary limitation is the narrow range of compatible substrates capable of producing high-quality LIG with adequate sensing performance. LIG is typically derived from PI and, to a lesser extent, from cellulose-based natural polymers. Moreover, the DLW process depends on a complex interplay of parameters, including laser wavelength, focal length, power intensity, scanning speed, and atmospheric conditions, all of which must be precisely optimized to ensure consistent material quality. The abundance of tunable variables makes standardization difficult, while substrate pre-selection and surface pre-treatment add further complexity. Mechanically, LIG prints tend to be brittle and exhibit poor adhesion, limiting their use as stand-alone films. Consequently, transfer to adhesive substrates, such as PDMS, is often required. To expand the sensing capabilities of LIG-based systems, additional printing or functionalization steps, such as material deposition or electrochemical modification, are frequently needed. Although ambient conditions introduce oxygen-containing groups that enhance sensing performance, they also reduce conductivity and degrade the graphitic structure, thereby restricting material customizability. Overall, DLW is a sophisticated technique that requires expert-level optimization and careful material selection, in contrast to the more accessible nature of SP and IJP. Future research should focus on improving LIG mechanical robustness, identifying low-cost and biocompatible substrates, developing dedicated, task-specific DLW systems for sensor production, and integrating ML or AI-assisted tools to streamline process optimization.

Future directions focus on overcoming current limitations, as this method remains in an early stage for printing plant sensors. A key technical priority involves developing in situ functionalization of LIG to selectively detect VOCs or chemical biomarkers released by plants, thereby minimizing post-printing modifications of LIG substrates. This could be achieved by pre-treating PI or other substrates with suitable precursors or introducing reactive gases into the printing chamber. To enhance LIG quality and resolution, pulsed laser techniques may be employed in place of continuous laser operation. This approach offers strong potential for large-scale, batch-to-batch sensor fabrication and can be integrated with established methods, such as IJP, SP, or spray coating. Additionally, the DLW process may serve as a post-printing strategy to increase the surface area of 3D-printed structures or electrodes, opening pathways for next-generation plant sensors.^223,224^ From a materials perspective, bio-based substrates, such as silk, wood^225^, cork^226^, and even plant leaves, offer a sustainable and low-cost platform for sensor fabrication. Additional opportunities include replacing PDMS through post-processing or print-transfer techniques using natural gels or stretchable biopolymers. Beyond sensing, LIG has been widely investigated in energy harvesting and storage systems, including triboelectric, thermoelectric, supercapacitor, and battery applications^213^. These approaches offer future opportunities for printing integrated, self-powered plant sensors. To date, only a limited range of dopants, such as iron, Ti_3_C_2_, and phosphorene, have been explored in DLW-based plant sensor development, whereas a broader spectrum of compositions has been studied in human health monitoring applications. Extending these well-established materials, including metal chalcogenides, MAX phases, and transition metal oxides, could enhance LIG performance for VOC detection. By implementing such strategies, the DLW method has the potential to advance or even transform plant sensor development.

### Aerosol jet printing for crop sensor development

**Fig. 6:**
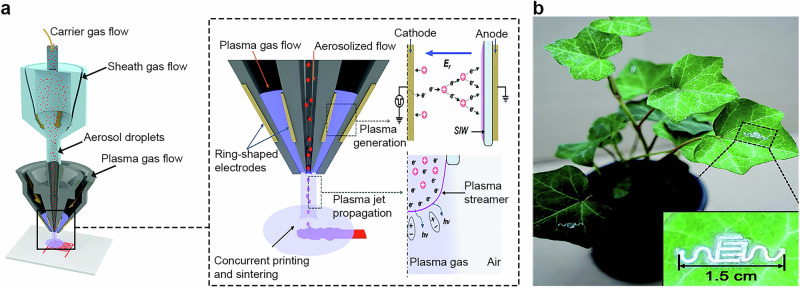
Aerosol jet printing (AJP) sensor for plant monitoring. **a** Schematic of the AJP consisting of an aerosol jet and a coaxial atmospheric pressure plasma jet for concurrent ink deposition and sintering. **b** The image of a pot of English ivy with a hydration sensor directly printed on its leaves. The inset shows a detailed view of the printed interdigitated silver electrode. The figure is adapted from Du, Y. et al.^57^, licensed under CC-BY 4.0 (https://creativecommons.org/licenses/by/4.0/).

AJP enables high-resolution, conformal deposition of functional inks on diverse substrates and curved surfaces^227,228^, making it well-suited for minimally invasive crop sensing^57,229^. Among the primary advantages of AJP are its ultrafine feature resolution^58,230^, broad material compatibility^231^, and ability to print conformally on soft or nonplanar surfaces^232^. These attributes reduce development cycles and enhance design flexibility in biointegrated electronics, aligning with the fabrication requirements of plant-attached and leaf-mounted sensors. A key limitation of metallic aerosol-printed electrodes is their requirement for post-printing thermal sintering^57^, which limits integration with temperature-sensitive biological tissues. A recent strategy overcomes this constraint by coupling an aerosol jet with a nonthermal atmospheric-pressure plasma jet in a coaxial head, which deposits and sinters simultaneously at near-ambient temperatures^57^ (Fig. 6a). The configuration envelops the aerosolized ink in a plasma-gas sheath, enabling in situ removal of organic stabilizers and densification during deposition while preserving fine structural features. This sophisticated approach was applied to print silver-based interdigitated hydration sensors directly on a living English ivy leaf (Fig. 6b), achieving high conductivity at biological interfaces and stable operation during irrigation and photosynthetic cycles. The printed interdigitated electrodes were used to monitor the leaf’s water content by measuring its electrical impedance, which varied as the tissue gained or lost moisture. Impedance spectra of hydrated and dehydrated leaves showed that water-rich tissues exhibited lower impedance across all frequencies, while dehydration caused ion channels to collapse and resistance to increase, especially at low frequencies where ionic conduction dominates. This behavior was further tracked in real time at 10 kHz under controlled changes in humidity, light, and irrigation, where impedance increased during dehydration and returned to initial values after re-watering, faithfully reflecting the plant’s natural hydration cycle. This simple but powerful method proved that the printed device could continuously track plant water dynamics without damaging the tissue, confirming that aerosol-jet-printed sensors can operate reliably on living leaves under realistic environmental fluctuations.

Despite its versatility and high resolution, AJP faces several technical and practical challenges that currently limit its broader industrial deployment. One of the primary issues lies in the complexity of ink atomization and aerosol transport. The process relies on the stable generation of micro-scale droplets, whose size distribution, solvent volatility, and carrier-gas dynamics must be carefully balanced to ensure uniform deposition^227^. Small deviations in temperature, flow rate, or nozzle geometry can result in beam deflection, overspray, or satellite droplets, all of which compromise line precision and surface morphology^233^. Maintaining this delicate equilibrium becomes increasingly demanding when printing multi-material or high-viscosity inks that exhibit complex rheological behavior.

Another challenge involves the stability and compatibility of functional inks. Although AJP supports a broader viscosity window than IJP, achieving reliable aerosolization without particle aggregation or nozzle clogging remains difficult. Nanoparticle-based inks, particularly those containing metals or carbon nanostructures, tend to agglomerate during aerosol generation, leading to inconsistent droplet delivery and poor film uniformity^234^. Furthermore, the high surface area of nanomaterials promotes rapid solvent evaporation within the nozzle, often resulting in the partial drying of particles and irregular deposition. Ensuring long-term print stability requires precise control of environmental parameters, such as humidity and carrier-gas composition, together with optimized ink formulation^58^.

Post-deposition processing presents additional difficulties. Conductive and semiconductive films produced by AJP typically require thermal or photonic sintering to achieve adequate electrical performance. These steps, however, can induce substrate deformation or crack formation, especially on flexible polymers or temperature-sensitive materials^235^. Achieving high conductivity at low processing temperatures remains one of the main technological hurdles for the method^236^.

Finally, reproducibility and scalability remain important concerns^231^. The dependence of AJP on multiple interdependent parameters, such as atomizer flow, sheath-gas focusing ratio, nozzle-substrate distance, and stage velocity, makes process optimization both time-consuming and system-specific. Minor variations in these parameters can produce significant differences in feature width, layer thickness, or surface roughness, which challenge large-scale standardization^227^. Moreover, the cost of specialized equipment, nozzle maintenance, and the need for skilled operation hinder its accessibility outside research laboratories.

The future development of AJP will be driven by advances that enhance process control, resolution, and material compatibility while reducing complexity and cost. Continued innovation in ink formulation will play a central role. The design of stable, low-temperature, and environmentally friendly inks with well-defined rheological behavior will broaden the palette of printable functional materials. In particular, the integration of nanostructured conductors, semiconductors, and dielectrics into multi-phase inks will enable the creation of high-performance sensor architectures suitable for flexible, wearable, and miniaturized electronics. The development of water-based or biodegradable inks will also support sustainable manufacturing and expand applications in biocompatible systems.

Another promising direction involves hybrid manufacturing approaches. Combining AJP with complementary additive techniques, such as inkjet, screen, or laser-based printing, will allow multi-material integration within a single platform. For instance, AJP can be used for fine conductive traces or microelectrodes, while SP provides thicker interconnects, and IJP enables selective deposition of functional coatings or sensing layers. These hybrid workflows will significantly expand design flexibility, reduce production time, and facilitate the development of complex device architectures with hierarchical structures.

Ultimately, the long-term vision for AJP lies in its transition from a versatile prototyping tool to a robust industrial manufacturing technology. Achieving this goal will require the convergence of advanced ink chemistry, precision fluid dynamics, real-time quality control, and scalable automation. By overcoming current barriers in reproducibility, cost, and process stability, AJP is poised to become one of the most adaptable and powerful additive manufacturing techniques for next-generation sensors and flexible electronics.

### Crucial physicochemical properties of printable inks for screen and inkjet printing

SP inks are typically thick, viscous pastes formulated to pass through a mesh stencil onto a substrate and to minimize bleeding. These inks commonly contain micron-sized particles (e.g., spheres or flakes) dispersed in a resin or binder matrix, with relatively low solvent content. Their high viscosity ensures the formation of thick, mechanically robust, and conductive films, making them well-suited for the printing of robust conductive patterns. SP inks tolerate a broader range of particle sizes and are less sensitive to ageing. In contrast, IJP inks are low-viscosity colloidally stable fluids formulated to be ejected through micron-scale nozzles. These inks typically incorporate nanoparticles to avoid clogging and ensure smooth jetting. To prevent agglomeration, such inks require careful stabilization and ideally should meet strict surface tension and viscosity criteria. IJP inks enable high resolution and precise patterning, but also commonly require post-processing (e.g., thermal or photonic sintering) to achieve desired properties, such as electrical conductivity. Detailed parameters are shown in Supplementary Table 2.

### Rheology

Rheology is a fundamental parameter in ink formulation, determining key aspects of the printing. When setting up a screen print with a novel ink, rheological measurements are the key to achieve fast print speeds while maintaining precision. The dynamic viscosity (*h*) and yield stress can be measured and designed to create an ink which passes through a mesh, leaves an even layer of defined thickness and resists spreading once deposited. The thixotropy and thermorheological properties of the ink can allow to improve the squeegee speed, pressure and angle in the process to work best with the novel ink. Viscosity for SP inks ranges from 1000 to 10,000 mPa s.^237^

In IJP, the optimal viscosity typically falls within the range of 1–20 mPa s, ensuring stable jetting performance^238^. A helpful metric in evaluating inkjet printability is the inverse Ohnesorge number (*Z*), which incorporates the Reynolds and Weber numbers and reflects a combined influence of key fluid properties, such as density, viscosity, and surface tension. While the commonly cited printable range is 1 < *Z* < 14^87^, this should not be interpreted as a rigid constraint in ink design. Especially in the case of nanomaterial inks, possible shear thickening and thinning effects should be taken into account and considered in the context of the target application. It has already been shown that water-based graphene inks are perfectly compatible with IJP technologies^148^, even though water itself has a *Z*-value of around 40 and therefore does not reach the parameters defined by the device manufacturers.

### Surface tension

Surface tension is another critical ink parameter that must be carefully optimized. While SP typically involves more viscous inks, surface tension (typically in 30–70 mN m^−1^ range)^239^ still plays an important role by affecting wetting of substrates (low surface tension improves wetting on low surface energy substrates), edge resolution, and stencil filling. In IJP, however, it significantly influences droplet formation and stability during the jetting phase; surface tension must therefore fall within a narrow optimal range of approximately 20–40 mN m^−1^, depending on the solvent system, to ensure stable jetting and consistent droplet behavior^146^. Beyond the printing process itself, surface tension is also essential at the ink-substrate interface, where it determines the droplet’s ability to wet, spread, and dry uniformly, enabling the formation of homogeneous and continuous films. Within the printing industry, it is generally considered that effective wetting is achieved when the ink’s surface tension is approximately 7–10 mN m^−1^ lower than the surface energy of the substrate^240^.

### Solid content

The solid content of an ink formulation, defined as the total mass percentage of nonvolatile components, plays a critical role in determining the final film thickness, morphology and overall rheological behavior of the ink. When printing the same volume of material, an IJP droplet typically dries to a film thickness on the submicron scale, whereas a SP layer can easily reach 10–13 μm. This inherent difference in deposited mass per layer is one of the reasons SP is widely favored for applications that demand high conductivity, such as printed interconnects and electrodes. Optimizing the solid content ensures sufficient loading of functional materials, leading to the formation of uniform films and sufficient conductivity with fewer print passes. SP, in particular, accommodates high solid contents (typically 40–70 wt%)^241^, enabling the use of formulations that are stretchable, thermally stable, or tailored for thick-film deposition. In contrast, IJP inks are limited to much lower solid contents (typically 5–30 wt%, although values as high as 40 wt% are also common, especially for silver IJP inks)^242^ to maintain low viscosity and prevent nozzle clogging, making them ideal for high-resolution, material-efficient deposition, especially when using expensive functional materials, such as gold.

### Particle size and shape

The size and shape of the particles affect the conductivity, film morphology and printability of the resulting structures. They also directly impact the cost and stability of the ink formulations. In conductive inks, silver-based particles are commonly used in various forms: microflakes and microspheres are typical contents of electrically conductive SP pastes, while silver nanoparticles are used in IJP inks. Optimizing particle size enables efficient percolation pathways and the formation of smooth films. Silver microparticles, for instance, are relatively inexpensive and less reliant on organic stabilizers. Experimental studies have shown that silver microparticles can be sintered at temperatures below 200 °C, achieving resistivities as low as 8.33 mW cm.^243^ In contrast, silver nanoparticles fuse rapidly at low temperatures due to their high specific surface area, strongly curved surfaces, and short diffusion lengths. As a result, treatments below 200 °C using thermal or photonic curing can produce highly conductive prints, with resistivities ranging from 3.45 to 8.0 mW cm^244,245^. However, these advantages come with challenges, as nanoparticles are more expensive and prone to agglomeration. Despite this, silver nanoparticle inks are routinely used with inkjet printheads, such as the Samba from Fujifilm. While utilizing such printers, precise control over particle size is essential to prevent nozzle clogging and maintain jetting stability. Although it is not strictly necessary to comply with the requirements of the printer manufacturers, which are often conservatively undersized, in general, the particle size should be no more than around 500 nm to achieve a stable and accurate printing process. Therefore, careful tuning of particle size distribution and shape is crucial for balancing conductivity, processability, and reliability in both SP and IJP technologies.

Once deposited, printed elements typically undergo a post-treatment or curing step to finalize their properties. This process can involve thermal/infrared curing, UV curing, and in advanced cases, laser or photonic sintering^246^. The primary goal is to remove residual solvents, initiate chemical reactions that harden the material, sinter metal particles together to form conductive paths or enhance adhesion between the printed material and the substrate^247^. When optimized, post-treatment results in maximal conductivity, durable printed features, and robust adhesion, even on thermally sensitive or flexible substrates. For example, higher curing temperatures can promote more efficient sintering of conductive particles, leading to lower resistivity. However, operating outside the optimal range (such as applying excessive heat or insufficient curing time) can lead to substrate deformation, incomplete sintering, poor adhesion, or mechanical failure under stress (e.g., bending, crack formation). Thus, careful tuning of the post-treatment conditions is critical to ensuring the long-term reliability and performance of printed electronic devices.

### Low-dimensional inks for crop sensor development

Among the most widely used conductive inks in the printing industry are formulations containing metal nanoparticles, particularly silver^234,248^ and gold^249,250^, or electroactive coordination compounds, such as Prussian blue^251,252^, due to their excellent electrical conductivity and printing ability^253^. These inks are primarily used for printing conductive traces and interconnects, essential for creating reliable electrical connections in printed electronic devices^254^. In contrast, for developing sensor platforms, particularly working electrodes (recognition part of the sensor), low-dimensional materials, such as graphene-based^255–257^, TMDs^258–260^, and MXenes^261–263^ have emerged as transformative candidates for next-generation printed sensors. Their atomically thin two-dimensional structures provide outstanding properties, including high conductivity, large specific surface area, broad chemical tunability, and mechanical flexibility. These properties are critical for fabricating compact, sensitive, and robust sensor systems.

Among them, graphene-based inks remain the most widely used in printable electronics due to their versatile functionality (surface chemistry), mechanical strength, and water solubility^127^. Their tuneable surface chemistry, which can be achieved via oxidation^264,265^, reduction^266,267^, addition^268,269^, substitution^270,271^, or heteroatom doping^272,273^ enables the design of tailor-made sensing platforms^274–276^. Although recent studies have demonstrated that graphene-based materials can form stable water-based ink formulations without additives^148,149,277^, the majority of functional inks still require the use of binders, surfactants, or stabilizers to prevent aggregation and sedimentation^126,127^. Another limitation of graphene derivatives is their inherently low conductivity^278^; for instance, nonconductive GO requires chemical or thermal reduction toward reduced GO (rGO) with a restored conductivity^279^. In addition, oxygen reduction must be precisely controlled, otherwise rGO is obtained with a low degree of functionalization, which is insufficient for immobilization of ions^280,281^ or (bio)molecules^282,283^. Beyond chemical modification and composite engineering, laser-based strategies have recently emerged as powerful tools to simultaneously reduce and nanostructure graphenic materials, enabling the fabrication of hybrid heterostructures with improved electrochemical performance (see “Direct Laser Writing” for details). For instance, laser-assisted decoration of GO films with noble metal nanoparticles has enabled the production of highly porous, conductive rGO-MNP films without binders or surfactants, showing great promise for miniaturized electrochemical sensors^284^. In parallel, laser-induced heterostructuring approaches have enabled the assembly of rGO-TMD hybrids on flexible substrates, forming 2D/2D architectures that leverage the conductivity of rGO and the catalytic properties of MoS_2_, WS_2_, MoSe_2_, or WSe_2_. The resulting sensors demonstrated nanomolar detection limits and high operational stability, positioning these techniques as promising alternatives for printed biosensing in agricultural and environmental settings^285^.

MXenes, a class of transition metal carbides or nitrides^286,287^, exhibit semiconducting or even metallic conductivity combined with hydrophilicity and diverse surface chemistry (less developed with respect to functionalized graphenes^288,289^), rendering them promising candidates for SP and IJP technologies^290^. However, key limitations hindering their widespread use are the susceptibility to oxidative degradation in aqueous or ambient environments, restacking during drying, and the need for toxic etchants (e.g., HF) during synthesis^290^.

TMDs, such as MoS_2_ and WS_2_, have intrinsic semiconducting properties with layer-dependent band gaps^291^, enabling their use in photoelectrochemical detection^292^, flexible field-effect transistors^293^, or light-responsive sensors^294^. Nonetheless, they often suffer from low exfoliation yields, ink instability, and re-aggregation of sheets during print deposition, leading to inhomogeneous film formation with poor electrical contact between layers, ultimately compromising device performance^295^. A targeted combination of these low-dimensional materials to create hybrid inks can eliminate undesirable shortcomings, as their complementary properties can provide improved sensitivity, selectivity, and stability^296–299^.

**Fig. 7:**
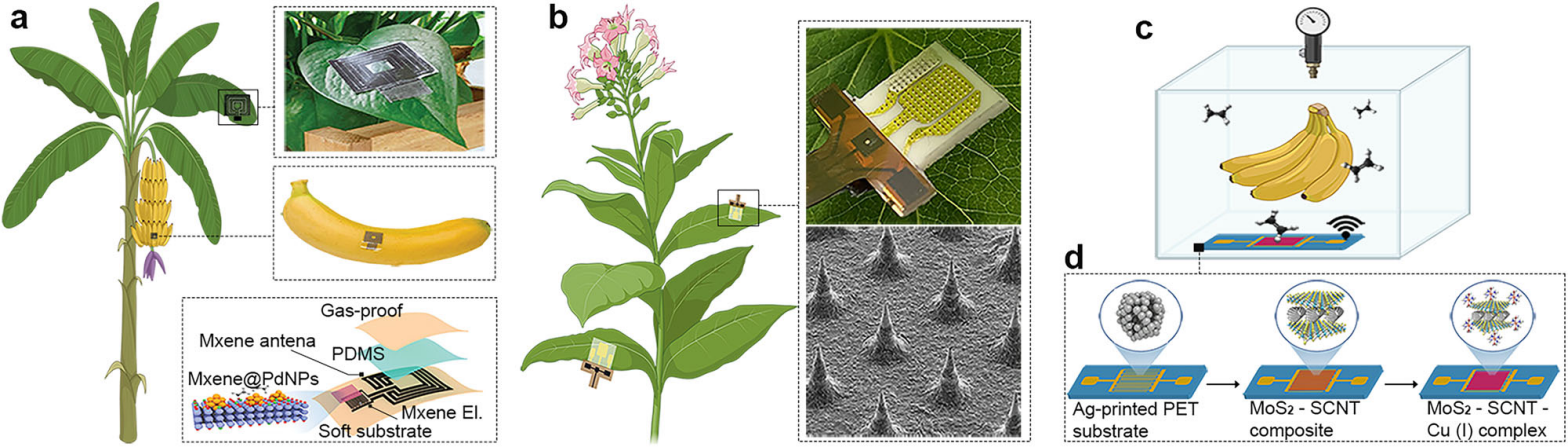
The use of low-dimensional inks for the development of sensors for crop sensing. **a** Photos of MXene-printed sensor as smart plant wearable tags on leaf and fruit, and fabrication of MXene-based sensor. **b** Photo of the microneedle sensor mounted on a plant leaf and SEM image of an Au-coated microneedle functionalized with chitosan-rGO hybrid. **c** Experimental setup for monitoring ethylene released by various fruit samples at room temperature using a wireless sensor. **d** Schematic of the process flow for the fabrication of sensors on a flexible substrate. Part **a** is adapted from Li, X. et al.^115^, licensed under CC-BY 4.0 (https://creativecommons.org/licenses/by/4.0/). Part **b** is adapted from Singh, N. et al.^302^, licensed under CC-BY 4.0 (https://creativecommons.org/licenses/by/4.0/).

Leveraging these unique properties, low-dimensional inks are used extensively in developing flexible, low-cost, high-performance sensors for agriculture^221,300–303^. Specifically, Ti_3_C_2_T_X_ MXene, due to its high electrical conductivity, hydrophilicity, and favorable rheological behavior, has been employed to develop a fully SP, wireless sensor platform for the detection of the volatile stress-related phytohormone ethylene on plant surfaces. While the additive-free MXene ink enables rapid, high-resolution printing of mechanically robust RF resonators (Fig. 7a), its intrinsic non-specificity toward target gases limits both selectivity and sensitivity. To overcome this, palladium nanoparticles (PdNPs) were immobilized on the MXene surface (Fig. 7a) to exploit the strong affinity of palladium for ethylene through π-adsorption mechanisms. The resulting MXene@PdNPs composite significantly enhanced selectivity for ethylene detection, achieving a limit of detection of 0.084 ppm and a pronounced signal response of 1.16% at 1 ppm. Due to its high flexibility, the sensor adapts to the fruit surface, enabling real-time monitoring of ethylene emissions correlated with ripening stages of apple, banana, mango, and kiwi, as examples, and offers wireless, battery-free readout with high reproducibility^115^.

Owing to its large surface area and chemical functionality, rGO has been utilized to develop a microneedle-based sensor platform for real-time monitoring of ROS in living plant tissues. However, the inherent aggregation tendency of rGO and its limited biocompatibility pose challenges for enzyme immobilization and uniform coating, key requirements for stable biosensing interfaces. To tackle these limitations, rGO was integrated with chitosan, a naturally derived, biocompatible polymer that enhances dispersion stability and provides functional groups for covalent enzyme attachment. This bio-hydrogel, enriched with horseradish peroxidase, formed the electroactive layer of the microneedle array, enabling in situ H_2_O_2_ quantification directly within plant leaves (Fig. 7b). The resulting sensor exhibited high sensitivity (14.7 μA/μM), a low detection limit (0.06 μM), and a rapid response time (see Fig. 7f, g), achieving robust electrochemical performance across a wide dynamic range (0.1–4500 μM) without the need for sample extraction or external instrumentation^302^. Although dichalcogenides exhibit excellent prerequisites for scalable sensor fabrication, such as low-cost input materials and compatibility with liquid-phase deposition techniques (solution processing), they often face challenges, including their intrinsically low electrical conductivity and limited selectivity toward target analytes. To address these constraints, a hybrid MoS_2_-based sensor platform has been engineered for ethylene monitoring in fruits (Fig. 7c). Integrating single-walled carbon nanotubes (SWCNTs) with exfoliated MoS_2_ enabled the formation of a conductive, porous network, which was subsequently coated with a copper(I) complex for wireless ethylene detection (Fig. 7d). This architecture combines the high charge mobility and conductive pathways provided by SWCNTs with the ethylene-selective molecular recognition properties of the Cu(I) complex, while MoS_2_ serves as a chemically modifiable semiconducting scaffold (Fig. 7d). The resulting thin-film (ca. 300 nm) sensor exhibits sub-ppm sensitivity to ethylene, with rapid and highly selective responses and minimal interference from other VOCs. Integrated onto a flexible substrate with low-power wireless transceivers, the platform enables real-time monitoring of ethylene molecules released from climacteric fruits under ambient conditions, showcasing its potential for scalable deployment in agricultural supply chains^301^.

### Biorecognition and stability

Printed electrodes in plant sensing are exposed to harsher and more variable environments than typical biomedical or wearable sensors. Their long-term performance depends on material selection, substrate–ink interactions, and environmental exposure (humidity, UV, and temperature). Plants introduce additional factors, such as surface chemistry variability, growth dynamics, and abrasion, all of which must be considered when designing the sensor system^304^.

Humidity is particularly critical, with environments routinely reaching 80–100% RH. In addition, plants are exposed to full-spectrum sunlight, including significant UV-A/UV-B radiation, and outdoor plant sensors may also experience temperature fluctuations from −10 to 40 °C. These parameters should be considered when designing sensor systems for plants. A typical printed plant sensor comprises a substrate and an ink. Substrate choice should reflect durability: polyimide (Kapton) generally withstands harsher conditions than PET, while TPU is, to date, the most common substrate for stretchable applications^305^. Challenges related to UV and humidity can be mitigated by encapsulating non-active parts of the sensor, for example, with Parylene-C, thin PDMS layers, or other protective coatings^306^. For the active layer, sintered metal inks tend to be more brittle than chemically cured metal inks, carbon-based or hybrid composites are frequently used to improve stability^307,308^. Conductive polymers, such as PEDOT:PSS are excellent for impedance and bio-interfacing, but PEDOT:PSS is humidity-sensitive. Crosslinking or encapsulation can substantially improve robustness^97^. Drawing on advances from human wearable electronics, especially encapsulation strategies and stable biointerfaces under sweat, motion, and environmental stress, can accelerate progress in plant biosensing. At the same time, plant systems present unique challenges and opportunities that will continue to drive new designs accommodating dynamic growth, high humidity, and outdoor exposure.

Across work on plants and agriculture, the long-term stability of biological recognition elements remains a major limitation^41,122,309^. Enzyme-based sensors generally remain active for only a few days under continuous use and last stored for a few weeks at 4 °C^302^. Their catalytic function rapidly declines at elevated temperatures, extreme pH, or in chemically complex plant environments^122^. Antibody immunosensors show similar constraints. They have poor tolerance to heat, UV, and proteolysis, and rarely remain stable beyond 6 weeks in cold storage^121,310^. Aptamers offer greater thermal and chemical resilience and broad pH ranges, but still remain vulnerable to UV damage and nonspecific adsorption of plant phenolics, polysaccharides, and proteins^311^. Consequently, even leading aptamer sensors achieve only months of shelf life and days of continuous operation, typically exhibiting progressive drift and fouling over multi-hour measurements^312,313^. This challenge has motivated increasing interest in alternatives, such as nanozymes and molecularly imprinted polymers, which offer far greater environmental resilience^303,314,315^. Systematic lifetime studies under real agricultural conditions (field exposure, diurnal cycles, biofouling) are largely absent and remain an open research gap.

### Complete system integration of printed sensors for agricultural applications

To progress printed sensors from the laboratory to real-world application (i.e., greenhouse, fields), electronic systems must also be developed that enable sensor integration, signal digitization, data processing, and wireless transmission. Given the overwhelming application of printed sensors for electrochemical monitoring, most sensors require integration with a potentiostat – an instrument that converts a chemical stimulus to an electrical signal – for signal readout and subsequent digitization. Potentiostats are typically relatively complex in design, using a trans-impedance amplifier (TIA) (to transduce signal from chemical to electrical) and a series of analog amplifiers (biosignals are typically at nano-scale, which are not large enough to surpass the resolution of analog-to-digital converters) and passive and active filters (electrical signals are very prone to noise induced from surrounding electromagnetic fields) to enable clear and repeatable sampling. For this reason, most demonstrations of printed electrochemical sensors offload circuit design and rely on commercial potentiostat modules, with PalmSens being one of the most popular providers^14,39,145,146^.

Due to recent advances in silicon technology that reduce the size and cost of analog electronics, however, it is now possible to create application-specific potentiostats that sacrifice versatility for light-weight, streamlined performance. These custom platforms can be sufficiently portable, low-cost, and integrate additional features, such as wireless data communication and data analytics to enable translation to real-world agricultural environments. Hossain and Tabassum developed a multiplexed plant monitoring system, integrating a voltage-divider and TIA circuit to measure physiological resistance-based parameters (temperature, strain, pressure, and relative humidity) and ethylene concentration^115^. An ESP32 microcontroller is not only used to enable reliable input waveform generation (needed for TIA operation) and output digitization, but also includes Wi-Fi integration to enable wireless data communication with an external PC. Importantly, a low-pass filter is added to the reference electrode input of the TIA circuit to eliminate high-frequency noise that often couples to analog pins within consumer electronics. With the development of this standalone sensing system, all of the sensor data recorded can be wirelessly transmitted to a smartphone application for easy and immediate access to experimental data by growers, saving time and resources that would be devoted to manual data collection.

Chen et al. used a similar approach to sample, store, and wirelessly transmit data from their printed ethylene sensor^301^. Rather than using electrochemical techniques for ethylene detection, however, the conductance of the sensor is recorded using a Wheatstone bridge: a resistance measurement circuit that uses a network of resistors to calculate the resistance (inversely proportional to conductance) of an unknown source (i.e., the sample). To guarantee high resolution for precise monitoring (0.0008%), a 24-bit analog-to-digital converter (>16 million voltage levels) is integrated instead of relying on the standard 8-bit (>1000 voltage levels) precision used in most microcontroller boards. Uniquely, this system is wirelessly powered using a small battery (3.5 × 2.5 cm). Removing communication and power cables creates a miniaturized form factor that can be placed in a variety of physically constrained environments (e.g., food containers).

Notably, Grell et al. integrated their gas-phase ammonium sensor into a custom-designed board to enable point-of-use impedance monitoring^38^. The circuit is designed as a modular unit that contains only the analog electronics necessary for impedance sensing, including a TIA for electrochemical transduction and a programmable gain network to prevent clipping of the signal during recording. The remaining computational tasks, such as digital-to-analog conversion of the input signal, output signal digitization, and serial data communication, are offloaded to a microcontroller board (Arduino DUE), which easily connects to the sensing module. This approach simplifies and streamlines the hardware development process, though the use of versatile plug-and-play computing boards increases the cost of production. Using this system, the data collected could then be combined with additional weather factors affecting soil nitrogen levels and analyzed using supervised ML approaches to predict ammonium as well as nitrate levels (without any additional sensing equipment) in soil up to 12 days in the future.

### Machine learning systems in smart agriculture

**Fig. 8:**
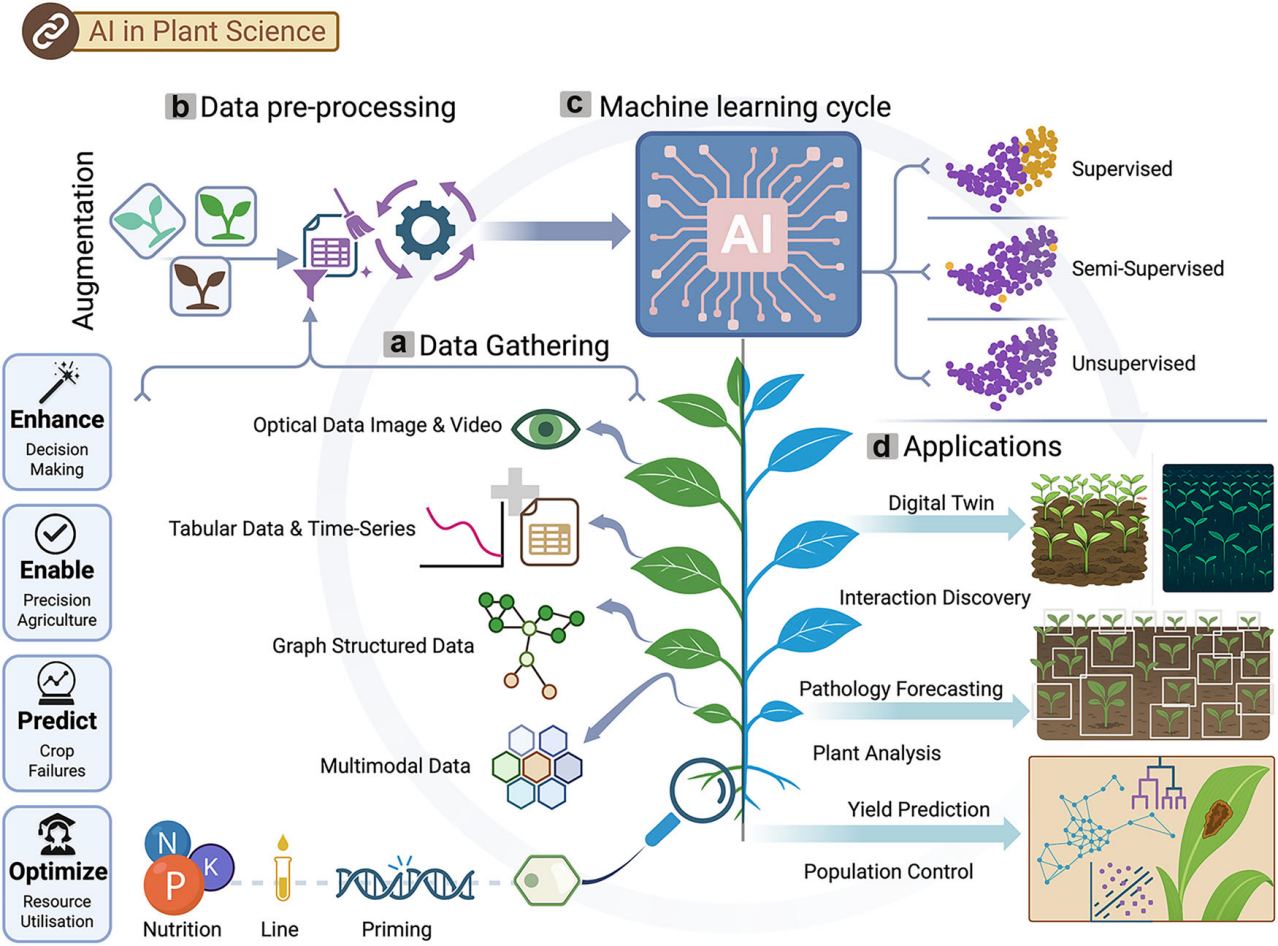
Machine learning pipeline connecting plant-centered sensing to agronomic decisions. Schematic overview of how machine learning operates across the plant-sensing workflow. **a** Heterogeneous data are collected from optical imaging and video, tabular and time-series measurements, graph-structured representations, and multimodal sensor streams, including plant-attached devices. **b** These signals undergo preprocessing and augmentation to clean, normalize, and expand the training distribution. **c** Supervised, semi-supervised, and unsupervised learning cycles develop both task-specific predictors and shared representations. **d** The resulting models support applications, such as digital twins, interaction discovery, pathology forecasting, plant analysis, yield prediction, and population management, enabling more accurate decisions, earlier stress detection, and more efficient use of resources in precision agriculture. Created in BioRender. De Diego, N. (2026) https://BioRender.com/nqq3tvk.

ML is transforming modern science by accelerating material design^316^, guiding drug discovery^317^, and enabling predictive modeling across disciplines^318,319^. It is also rapidly reshaping plant science, where ML integrates diverse streams of sensor, image, and omics data to advance biological understanding (Fig. 8). The diagram outlines how ML connects data gathering, preprocessing, and model learning to real-world applications, such as stress diagnosis, yield prediction, and digital-twin simulations. By closing the loop between continuous sensing and decision-making, ML now underpins precision agriculture and the predictive monitoring of crop health. Among its most immediate impacts is the ability to interpret the vast amount of data generated by emerging printed and wearable sensors, turning continuous physiological measurements into quantitative insights. This convergence between smart sensing and intelligent modeling defines the modern frontier of crop-health monitoring.

Crop-health monitoring increasingly combines continuous, plant-attached sensors with ML to turn noisy, multimodal signals into interpretable measures of stress type, severity, and timing. Across plant science, supervised learning (models trained on labeled examples) has become the mainstay for stress recognition and trait prediction, while unsupervised and representation learning (methods that infer patterns directly from unlabeled data) reveal structure in high-dimensional data and guide more stable, generalizable models. ML thus forms the analytical bridge between complex sensor signals and biological interpretation, allowing systems to move from raw measurements toward physiological meaning.

Plant-mounted sensing platforms now routinely classify stress states with compact models. An implantable microneedle array, for example, maps electrophysiological time series to stress classes using gradient-boosted (XGBoost) and tree-based algorithms^320^. An activatable near-infrared II fluorescent nanosensor, paired with supervised classifiers, distinguishes biotic from abiotic stress in vivo^42^. A multimodal leaf-patch wearable (MapS-Wear) learns spectral–microclimate relationships for early stress diagnosis through a hybrid unsupervised/supervised pipeline^321^. Physiological timing, such as internal circadian phase, a regression target critical for intervention planning, can also be estimated from transcriptomic profiles using ensemble neural networks trained for cross-condition generalization^322^. Together, these studies show how supervised frameworks convert sensor dynamics into actionable biological insight, enabling earlier and more reliable diagnosis.

In image-based phenotyping, ML supports field pipelines that use object detectors and semantic segmenters to localize organs, quantify canopy structure, and associate these features with morphological or physiological traits. AMULET integrates DeepLab-based segmentation, temporal forecasting (SimVP), and explainable ML (TorchGrad) to predict growth and stress traits hours to days in advance from RGB imagery^43^. PlantServation extends this idea to long-term outdoor monitoring, using ML-based phenotyping to quantify seasonal pigment shifts and genotype-specific differences^51^. Together, these approaches transform continuous image streams into biologically interpretable trajectories, linking field imaging to developmental physiology.

Unsupervised and representation learning further organize the complexity of plant data. Dimensionality-reduction and batch-integration methods, such as UMAP, t-SNE, and graph-based clustering underpin recent single-cell atlases that map immune cell states (PRIMER) and root adaptations to soil stress. These atlases contextualize sensor outputs and reveal pathways underlying stress phenotypes^323,324^. In sequence-to-function modeling, deep convolutional networks (CNNs) infer cis-regulatory logic directly from plant genomes, providing mechanistic priors that inform downstream trait and stress models^325^. Even classical methods, such as kernel principal component analysis and clustering, remain valuable in wearable-sensor pipelines, where they compress raw signals before supervised inference. Together, these strategies impose order on high-dimensional biology, allowing models to learn structure rather than noise.

Interpretability has also become a defining goal in plant ML. Feature-attribution techniques (e.g., SHAP, LIME, and integrated gradients) and saliency mapping now connect model predictions with physiological processes in both multi-omics trait prediction^326^ and image phenotyping^43,51^. Knowledge transfer across species and data-limited crops represents a parallel frontier from circadian-time ensemble models that generalize beyond *Arabidopsis*^322^ to strategic roadmaps advocating transfer learning and ML-assisted phenomics for orphan crops^327^. These efforts highlight that transparency and reusability are as important to progress as accuracy itself.

Printed and flexible devices generate the data, and ML translates those data into decisions. Together, they define a sensor-to-decision continuum that spans classification, regression, calibration transfer, and drift correction^328^. The recent literature outlines a practical taxonomy: supervised classification and regression for early stress typing and timing across wearable^320^, optical^42,43^, and omics data^326^; supervised segmentation and detection for field phenotyping^43,51^; and unsupervised representation learning that structures biological variation and guides model design^323,324,329^, with mechanistic ML on enzymes extending these ideas beyond sensors^330^. In this view, ML is not an accessory to sensing but the computational heart that turns printed signals into interpretable plant intelligence.

Printed and field-deployable crop sensors will continue to generate torrents of biological signals that rarely live in flat Euclidean space. Many of these data occupy curved or constrained manifolds, meaning that models encoding geometry (symmetries, graphs, and geodesics) are best positioned to generalize from lab to field^331^. This is not a theoretical curiosity: neural population activity can organize on a torus, reminding us that real biological processes often evolve along low-dimensional, manifold structure^332^.

Yet common visualization tools, such as t-SNE and UMAP, can fracture or over-separate structure, producing alluring but misleading patterns. Recent diagnostics reveal that neighbor-embedding maps contain intrinsic discontinuities and that hyperparameters, including perplexity, can fabricate spurious sub-clusters. Before drawing biological conclusions from such embeddings, guardrails and quantitative quality scores are essential^333^.

As ML models move closer to field decision-making, whether flagging stress, scheduling irrigation, or recommending interventions, explainability must become a first-class design principle. Reliable interpretation requires stability and faithfulness tests, human and lab-in-the-loop validation, and systematic cross-checks to ensure that apparent drivers are not artifacts of data or modeling^334^. Equally important, biological data streams often rely on labels that are scarce, biased, or variable across environments. This argues for greater use of unsupervised, contrastive, and continual learning to exploit unlabeled, evolving sensor inputs and to stress-test conclusions across contexts^335^.

In short, the field should lean into geometry-aware priors that respect biological structure332, diagnose and de-risk embeddings before storytelling333, and rebalance efforts from label-hungry supervision toward interpretable, self-supervising frameworks anchored in rigorous sensitivity checks. This is how ML will deliver durable, biologically faithful value to printed and flexible sensing for crop health.

### Future visions of printed sensors in smart agriculture

#### Low-dimensional smart inks

The future of printed sensors in smart agriculture will be driven by developing novel low-dimensional smart inks. These advanced materials offer uniquely tunable electronic, optical, and chemical properties, making them especially suitable for integration into miniaturized, high-sensitivity sensor platforms. The focus for further innovation will be on the formulation of printable, biocompatible, and water-dispersible compounds that are environmentally degradable, aligning with green chemistry principles and enabling sustainable deployment in open-field and greenhouse conditions. In this respect, graphene derivatives, MXenes, metal-organic frameworks, covalent organic frameworks, and nanocomposites based on these nanomaterials offer promising properties for these applications.

### Biodegradable and eco-friendly substrates

With the increasing deployment of sensors in agriculture, biodegradable and organic substrates are emerging as a major future trend. Traditional plastic-based electronics pose environmental risks when used in open-air systems or greenhouses, especially in large-volume, single-use formats. To address this issue, future printed sensors will increasingly rely on sustainable substrates, such as cellulose paper, tissue, or starch-based films that degrade naturally without harming soil microbiomes or plant systems. Combined with water-based inks, biodegradable substrates will support the development of waste-free sensor platforms for precision agriculture, which aligns with global sustainability initiatives.

### Multi-modal and multiplexed sensing platforms

Future agricultural diagnostics will increasingly rely on multimodal sensing platforms that integrate electrochemical, optical, mechanical, and thermal sensing capabilities into a single device. This convergence enables simultaneous monitoring of diverse physiological and environmental parameters, such as ion fluxes, stress-related phytohormones, ROS, VOCs, and temperature. Moreover, future platforms will incorporate multiplexed sensing architectures, including sensor arrays with multiple working electrodes or distinct signal transducers, capable of monitoring several analytes simultaneously in a localized environment. Such designs will improve the sensor efficiency and reduce system complexity, fabrication time and cost. Multi-modal and multiplexed platforms further allow cross-validation between modalities (e.g., correlating temperature shifts with oxidative stress), enhancing diagnostic accuracy and robustness against signal drift. These advanced systems will be essential in transitioning from single-analyte detection to an integrated, comprehensive plant health monitoring, increasing precision in agriculture with rich, real-time datasets.

### Energy harvesting and self-powered systems

A significant limitation to the widespread adoption of printed agricultural sensors is their dependence on external power sources or the need for frequent battery replacement. As a future trend, integrating energy harvesting and self-powered systems will be essential to achieve autonomous, maintenance-free sensor networks. New energy harvesting strategies based on solar energy, printed thermoelectric generators (exploiting temperature gradients between plants and air), and piezoelectric films (harvesting energy through mechanical vibrations from the wind). Combining these technologies with printed electronics with very low power consumption will allow fully autonomous sensing units to operate continuously in the field and support real-time data collection with minimal environmental impact and without external intervention.

### Artificial intelligence-enhanced data processing

Artificial intelligence (AI) is rapidly transforming the landscape of agricultural sensing, both in the development of advanced sensor materials and in the interpretation of complex data from multiple sources. On the materials side, ML algorithms are being used to predict key physical and chemical properties, control design, and accelerate the discovery of high-performance materials, significantly shortening experimental cycles. At the system level, ML enables the integration of multimodal sensor arrays, allowing for the seamless fusion and evaluation of multiple types of monitoring (e.g., electrochemical, optical, and thermal), thereby improving and accelerating crop risk prediction. Through intelligent filtering, signal correction, and multidimensional pattern recognition, AI tools can detect fine morphological and physiological changes in plants before visible symptoms appear. As sensor networks grow in scale and complexity, AI will be essential for real-time stress detection, nutrition optimization, and predicting plant growth scenarios. In the future, the integration of AI with virtual and augmented reality (VR/AR) platforms will enable intuitive interfaces for farmers that link sensor analysis with human–machine interaction in the field, facilitating faster decision-making and reducing the impact of environmental fluctuations on plant yield.

### Wireless data transmission

As sensors become increasingly widespread, miniaturized, and integrated into plant tissues and growth environments, their utility depends not only on sensitivity and selectivity but also on the ability to transmit data wirelessly and reliably in real-time. Next-generation sensors will be closely coupled with low-power wireless communication protocols, including Bluetooth or near-field communication, to enable seamless connectivity across sensor networks in greenhouses and open-field environments. The integration of these communication interfaces with self-powered sensor units and cloud-based AI systems will enable fully autonomous sensing pipelines that include data collection, transmission, and remote analysis. Such interconnections will be essential for implementing real-time feedback mechanisms in smart agriculture, where signals from leaf sensors, soil probes, or environmental monitors can autonomously drive irrigation, nutrient delivery, or climate control.

### Smart greenhouse architecture for precision agriculture

As fertile land continues to decline globally due to urban expansion, climatic pressures, and soil degradation, smart greenhouses are becoming essential infrastructures for crop production. These systems with autonomous controlled environments offer an ideal platform for regulating light, humidity, temperature, and nutrient supply through real-time AI-driven analysis. By integrating electrochemical and optical sensors throughout the greenhouse environment (e.g., soil, leaves, stems, fruits, or greenhouse construction), ML evaluation systems can generate robust datasets crucial for the precise and early detection of plant stress. Printed technologies, in particular, can facilitate scalable monitoring in environments, such as greenhouses (high volume is required to achieve sufficient spatial resolution), because of their significantly low cost of fabrication and easy customization. Combined with the continually decreasing cost and size of low-power electronics, printed sensors can be easily integrated with compact computing modules to enable continuous monitoring of key physiological parameters, on-board data processing, and automated wireless transmission to cloud-based servers for secure and easily accessible storage. Such devices may act as “nodes” within an IoT network, collecting real-time feedback that can be instantly translated using AI to provide unique physiological insights (e.g., nutrient dynamics, stress markers) and accessed directly on demand or as part of a decision support system for agricultural intervention. The use of printed sensors also adds a whole new modality of data (i.e., electrochemical) to traditionally image-based training datasets for predictive analytics through AI implementation. Unique nonlinear signatures can be extracted from deep learning models to provide early/near-immediate indicators of physiological change long before visual symptoms appear. Incorporating such data from printed devices could, therefore, dramatically reduce the latent period between physiological intervention and predictive output, resulting in timelier and, ultimately, more efficient resource utilization. The combination of self-powered sensors, energy harvesting technology, and AI-driven decision-making systems will enable autonomous operation, where sensor feedback dynamically regulates irrigation, ambient temperature, and fertilization, while facilitating continuous monitoring of pest infestation. Future greenhouses will also be equipped with robotic interface units capable of targeted interventions to support fully autonomous smart farming.

### Outlook

The convergence of materials science, printing technologies, and plant biology has redefined the development of smart sensors for precision agriculture, offering unprecedented potential for either noninvasive continuous monitoring or structurally integrated sensing, depending on the printing approach employed. Printing techniques, such as SP, IJP, 3DP, and DLW, have each contributed unique capabilities to the field, from high-throughput production to microscale patterning. Likewise, the integration of advanced low-dimensional materials, including graphene derivatives, MXenes, and TMDs, has elevated the sensitivity, flexibility, and functionality of printed sensors. Yet, despite these advances, critical limitations persist, ranging from constraints in ink formulation and accessibility of printing hardware to issues of durability, environmental stability, and real-world integration. The gap between laboratory innovation and field-deployable systems remains a significant barrier, underscoring the need for robust material formulations, platform miniaturization, sustainable substrates, and streamlined system integration. While current technologies have laid a strong foundation, achieving reliable, field-ready sensors still demands cross-disciplinary refinement at both the material and system levels.

The future of printed agricultural sensing will be defined by the seamless integration of materials design, advanced manufacturing, and intelligent data systems. To fully realize this potential, the field must move toward hybrid ink formulations that optimize both performance and printability, alongside the development of modular, portable printing platforms that bridge the gap between laboratory innovation and on-field deployment. Expanding the commercial availability of functional inks tailored to various printing technologies will be a major enabler, broadening access and accelerating adoption. Inks compatible with standard office printers offer significant potential to democratize this technology, enabling accessible entry points for researchers, innovative growers, and decentralized applications. At the same time, establishing robust validation protocols and standardized testing frameworks is crucial to ensure reproducibility, regulatory compliance, and scalability across diverse agricultural settings. The rise of ML offers further transformative potential, not only for interpreting complex, multidimensional sensor data but also for optimizing material formulations, predicting long-term behavior, and enabling autonomous, self-correcting sensing platforms. In this vision, printed sensors will evolve into resilient, adaptive systems capable of detecting early plant stress responses, implementing precise interventions, and managing resources efficiently. By merging sustainable, environmentally friendly materials with smart diagnostics and predictive analytics, printed technologies can play a pivotal role in securing global food systems and advancing sustainable agriculture. The true challenge now lies not in innovation alone, but in translating that innovation into reliable, accessible tools that operate effectively under real-world agricultural field conditions, where the future of farming will ultimately be shaped.

In conclusion, we highlight emerging trends, noting that the transition of printed and biodegradable sensors from laboratory prototypes to field tools will depend on their robustness, stability, low maintenance requirements, and seamless data connectivity. By addressing these practical requirements and aligning device design with real agricultural conditions, sustainable plant-wearable devices can become practical components of precision agriculture, supporting more resilient crop production.

## Supplementary information

Supplementary Information
