# Supplementary material for: Printing technologies for monitoring crop health
Source: Nat Commun. 2026 Jan 24;17:2009. doi: 10.1038/s41467-026-68778-6 (PMC12936174; doi:10.1038/s41467-026-68778-6)
Supplement: Supplementary file 1 — Supplementary Information [file 41467_2026_68778_MOESM1_ESM.pdf]

Printing technologies for monitoring crop health

Panáček *et al.*

Supplementary Table 1. Overview of printing methods and their key parameters.

Printing technique	Standard resolution [a]	Manufacturing time [b]	Price of inks/resins	Ink losses [c]	Scalability[d]	Compatibility with substrates and inks/resins [e]		Versatility [f]
						**Inks/resins**	**Substrates**	
Screen printing	50–150 µm	Fast	Low	High	High	Ag flakes, carbon (graphite/graphene	Flexible plastics (PET, PI), paper/cellulose, textile	High
Inkjet printing	20–40 µm	Moderate	High	Low	Moderate	Ag NPs, PEDOT:PSS, water-based graphene, biomolecules	Leaf/stem, flexible films, hydrogels, transfer media	High
3D printing	100–500 µm	Moderate	Moderate	Low	Moderate	Photopolymer resins, thermoplastics, biodegradable filaments	Result itself is a structural platform which can be further processed	Good
Direct laser writing	0.1–1 µm	Slow	High	Low	Limited	Inkless formation of laser-induced graphene	Polyimide, polydimethylsiloxane, carbon-based polymers	High
Aerosol jet printing	10–50 µm	Moderate	High	Low	Moderate	Metal and carbon inks	Leaf, textile, polymers	Very high

[a]Typical lateral feature size (printed line width or gap) achievable under standard settings: single layer, single pass, vendor-recommended conditions.

[b]End-to-end process time per unit area under standard settings, including essential post-processing.

[c]Qualitative assessment of how much of loaded material is lost, i.e. not utilized in the printed features. Actual utilization is process- and setup-dependent (ink formulation, mesh/nozzle, cleaning protocol, process length) and can improve with reclaim/reuse workflows.

[d]Ease of increasing throughput and area under standard settings, considering parallelism, coverage dependence, and typical post-processing.

[e]Representative materials and substrates currently available for each technique, selected for plant/crop monitoring use cases. Entries reflect routine practice and are not exhaustive.

[f]Breadth of usable materials, substrates or geometries, and patterning flexibility.

Supplementary Table 2. Summary of the crucial physicochemical properties of screen printing (SP) and inkject printing (IJP) inks.

Parameter	Optimal range		Role in printing	Advantages of optimization	Challenges outside optimal range
	SP	IJP			
Rheology	η=1,000–10,000 mPa·s; shear thinning fluid	η=1–20 mPa·s	Controls droplet ejection and formation, layer thickness, and resolution	Stable jetting, high resolution, minimal spreading, consistent deposition	Unpredictable jetting, satellite droplets, bleeding, uneven thickness, print failures
Surface tension	30–70 $mN \cdot m^{-1}$	20–40 $mN \cdot m^{-1}$	Affects droplet formation and shape, substrate wetting, film continuity	Continuous jetting, uniform film coverage, strong adhesion, minimal overspreading	Poor wetting or overspreading, nozzle wetting/flooding, poor adhesion
Solid content	5–40 wt.%	40–70 wt.%	Determines film conductivity, thickness, and morphology, affects ink's rheology	High conductivity, fewer print passes, controlled drying shrinkage	High viscosity, agglomeration, clogging, sedimentation, print defects, poor conductivity
Particle size	Microparticles, microflakes	Commonly nanoparticles	Influences percolation, conductivity, and sintering behavior	Low-temperature sintering, high conductivity, smooth films, no clogging	Aggregation, increased viscosity, nozzle clogging, rough layers
Post-treatment	Thermal curing, photonic/laser sintering	Thermal curing, photonic/laser sintering	Removes solvent, sinters particles, improves adhesion, durability and conductivity	Maximal conductivity, durability, robust adhesion, substrate compatibility	Substrate or pattern damage, incomplete sintering, poor adhesion and mechanical properties
